# Cooperative Al(Salen)-Pyridinium Catalysts for the Asymmetric Synthesis of *trans*-Configured β-Lactones by [2+2]-Cyclocondensation of Acylbromides and Aldehydes: Investigation of Pyridinium Substituent Effects

**DOI:** 10.3390/molecules17067121

**Published:** 2012-06-12

**Authors:** Patrick Meier, Florian Broghammer, Katja Latendorf, Guntram Rauhut, René Peters

**Affiliations:** 1 Institut für Organische Chemie, Universität Stuttgart, Pfaffenwaldring 55, 70569 Stuttgart, Germany; Email: meier@theochem.uni-stuttgart.de (P.M.); florian.broghammer@oc.uni-stuttgart.de (F.B.); katja.latendorf@oc.uni-stuttgart.de (K.L.); 2 Institut für Theoretische Chemie, Universität Stuttgart, Pfaffenwaldring 55, 70569 Stuttgart, Germany; Email: rauhut@theochem.uni-stuttgart.de

**Keywords:** aluminum, cooperative catalysis, ion pair catalysis, ketene, 2-oxetanone, pyridinium

## Abstract

The *trans*-selective catalytic asymmetric formation of β-lactones constitutes an attractive surrogate for *anti*-aldol additions. Recently, we have reported the first catalyst which is capable of forming *trans*-β-lactones with high enantioselectivity from aliphatic (and aromatic) aldehyde substrates by cyclocondensation with acyl bromides. In that previous study the concepts of Lewis acid and organic aprotic ion pair catalysis were combined in a salen-type catalyst molecule. Since a pyridinium residue on the salen periphery is essential for high *trans*- and enantioselectivity, we were interested in the question of whether substituents on the pyridinium rings could be used to further improve the catalyst efficiency, as they might have a significant impact on the effective charges within the heterocycles. In the present study we have thus compared a small library of aluminum salen/bispyridinium catalysts mainly differing in the substituents on the pyridinium residues. As one result of these studies a new catalyst was identified which offers slightly superior stereoselectivity as compared to the previously reported best catalyst. NBO calculations have revealed that the higher stereoselectivity can arguably not be explained by the variation of the effective charge.

## 1. Introduction

β-Lactones (systematic name 2-oxetanones), are attracting the interest of scientists for mainly two reasons: (1) a number of natural and synthetic β-lactones are known to act as specific enzyme inhibitors [1,2,3,4]. Tetrahydrolipstatin (Xenical^®^, orlistat), for instance, is used for the treatment of obesity and has recently received renewed attention due to the finding that it is capable of specifically inhibiting fatty acid synthase (FAS-TE), an approved drug target for cancer treatment [5,6,7]; (2) as a result of their inherent ring strain [8] β-lactones are useful synthetic building blocks. Ring opening with hard nucleophiles offers the possibility to a divergent access to aldol products [9,10,11,12,13], whereas treatment with soft nucleophiles can be utilized to synthesize β-functionalized carboxylic acid derivatives [9,10,12]. In both cases the stereoinformation of the β-lactone ring can be completely transferred into the ring opening product and the acyl-oxygen bond cleavage with hard nucleophiles typically proceeds with retention of configuration. *Cis*- and *trans*-configured β-lactones thus behave as masked and activated *syn*- and *anti*-aldol equivalents, respectively.

The catalytic asymmetric formation of ester or amide aldol derivatives via β-lactones is not only attractive for the high divergency of accessible aldol structures, but also for the high atom-economy of the overall process, since no preformation of silylketene acetals or related nucleophiles is necessary [14]. Instead an acyl halide is usually used to generate a ketene *in situ* as a reactive intermediate by dehydrohalogenation induced by a base [15,16,17,18,19]. The ketene then undergoes a cycloaddition with an aldehyde, either catalyzed by a Lewis acid [20,21,22,23,24,25,26,27,28,29] or by a Lewis base [30,31,32,33,34,35,36,37] or by a combination of both [38,39,40,41,42]. Particularly appealing appears the development of *trans*-selective catalytic asymmetric β-lactone formations as a surrogate for *anti*-aldol additions, since for the latter reaction type only few efficient catalytic asymmetric protocols exist to date [43,44,45,46].

Unfortunately, the catalytic asymmetric [2+2] cycloaddition approach of ketenes and aldehydes provides in general only *cis*-diastereomers as the major products [47,48,49]. In 2008, Calter *et al.* described the first catalytic enantioselective access to *trans*-configured β-lactones by a formal [2+2] cycloaddition [39]. However, this method is so far limited to the use of aromatic aldehydes, whereas the large majority of bioactive compounds such as tetrahydrolipstatin carry an aliphatic chain at the 4-position of the 3,4-disubstituted 2-oxetanone core [50,51,52].

**Scheme 1:**
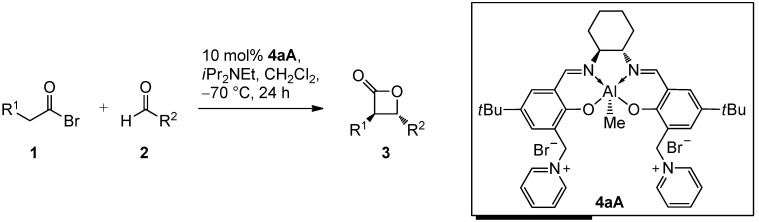
Application of bispyridinium Al-salen complex **4aA** for the *trans*-selective catalytic asymmetric synthesis of 3,4-disubstituted 2-oxetanones **3** [53,54].

Recently, we have reported a conceptually new approach which is applicable to both aliphatic and aromatic aldehydes [53,54]. Aluminum salen complexes carrying aprotic organic ion pairs in the periphery of the salen core were investigated. The most useful described catalyst **4aA** in terms of enantioselectivity and reactivity was equipped with two pyridinium bromide functionalities connected via benzylic methylene linkers to the *ortho*-position of the phenolate O atoms (3/3'-position, Scheme 1). 

**Scheme 2:**
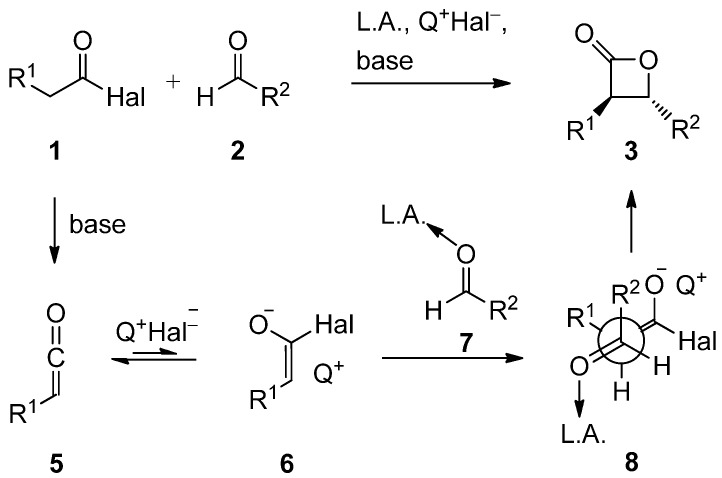
Rationale of the catalyst design for the *trans*-selective catalytic asymmetric synthesis of 3,4-disubstituted 2-oxetanones **3** [54].

By the choice of a Lewis acid with only one available coordination site a cyclic Zimmerman-Traxler-type transition state leading to the *cis*-configured product would be avoided. Our catalyst combines the cooperative action of a Lewis acid and of an aprotic onium halide ion pair Q^+^X^−^ (in the case of **4aA** pyridinium bromide), the latter being arguably used to generate the acyl halide enolate **6** in the catalyst sphere (Scheme 2). Q^+^ might stabilize the otherwise unstable enolate [55,56,57,58] by ion pair formation to increase the lifetime of the enolate species and to direct the enolate to the aldehyde-Lewis acid complex **7** via an open transition state **8** adopting a staggered conformation around the generated C–C bond [54]. In the reactive conformation the disubstituted enolate C-1 atom should be oriented *gauche* to the aldehyde function’s H atom and the C-2-enolate-H atom would be expected to direct toward the sterically demanding Lewis acid-ligand complex, so as to minimize repulsive interactions. The initial aldol adduct could then cyclize to form the heterocyclic product **3**. 

**Figure 1:**
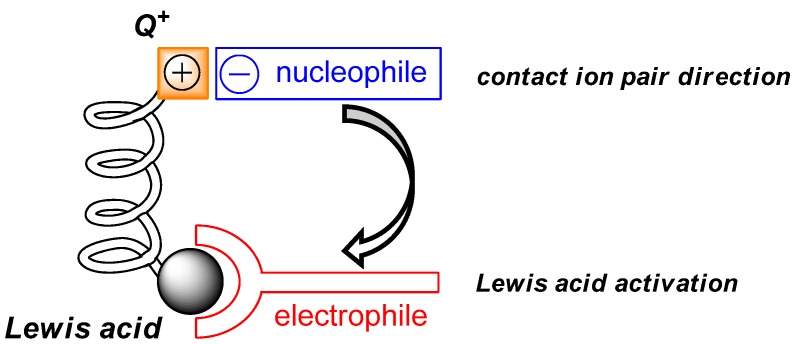
Cooperative action of a Lewis acid and an aprotic organic ion pair within a single catalyst entity: the Lewis-acid serves to activate an electrophile, whereas the *in situ* generated anionic nucleophile forms an ion pair with an aprotic organic cation (Q^+^), which stabilizes and directs the nucleophile towards the electrophile [53,54].

The general catalyst principle, utilizing the cooperative action of a Lewis acid and an aprotic organic ion pair, is illustrated in Figure 1.

Salen ligands were chosen due to their modular nature and their ready and rapid accessibility [59,60,61]. As a result of the proposed stabilizing electrostatic interaction of Q^+^ and the acyl halide enolate **6**, the charge density of Q^+^ was expected to play a decisive role on the reaction outcome, in particular in terms of *trans*/*cis*-diastereoselectivity and enantioselectivity, because neutral substituents at the 3/3'-position lead predominantly to the undesired almost racemic *cis*-diastereomer [53,54]. 

**Figure 2:**
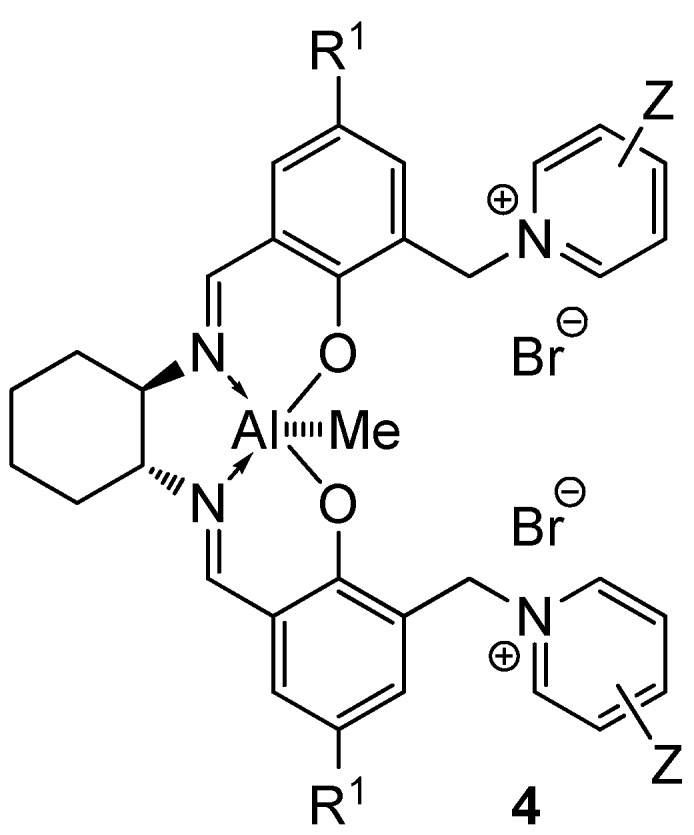
Structure of the investigated aluminum-bispyridinium catalysts **4** carrying different residues R^1^ and pyridinium substituents Z.

In our continuing efforts to utilize ketenes [62,63,64,65] and related reactive intermediates [66,67,68,69,70,71] for a rapid and practical access to chiral building blocks we have therefore investigated a series of Al-salen catalysts **4** (Figure 2) carrying different substituents Z on the pyridinium moiety (acting as Q^+^) to study electronic effects on the catalyst performance. The results are compared to NBO calculations which have revealed the charge distribution within the pyridinium rings.

## 2. Results and Discussion

### 2.1. Ligand Preparation

In our previous studies we found that a major limitation of catalyst **4aA** results from its relatively low solubility at low reaction temperatures. For that reason we were also interested in derivatives in which the phenolic *t-*Bu-substituents have been formally replaced by *n*-pentyl groups in order to increase the rotational freedom of the alkyl substituents R^1^ and hence the catalyst solubility. To investigate the steric influence of the R^1^ substituent on the stereoselectivity, R^1^ = Me was also studied. 

**Scheme 3:**
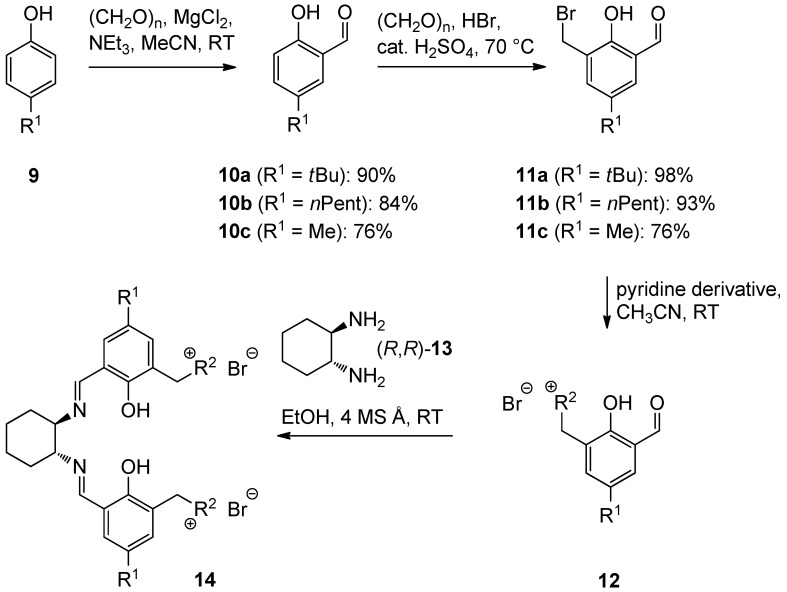
Synthesis of a salen ligand library **14** with different residues R^1^ and R^2^.

Based on our previously established protocol [53,54] the ligands **14** could be prepared via a general 4-step procedure (Scheme 3). Phenols **9** were formylated with paraformaldehyde in the presence of NEt_3_ and MgCl_2_ [72,73]. A subsequent bromomethylation of **10** with paraformaldehyde, HBr and catalytic amounts of sulfuric acid gave benzyl bromides **11** in high yields [74]. The latter were then treated with different commercially available pyridine derivatives in a nucleophilic substitution reaction in acetonitrile (Table 1). The pyridinium salts **12** could be isolated and purified by precipitation and washing with diethyl ether. Nucleophilic substitution proceeded smoothly in most cases. Only the 4-Cl derivative **12bH** was prone to releasing the starting pyridine in the back reaction (entry 11). This is ascribed to the electron-withdrawing character of the Cl-substituent, which reduces the nucleophilicity of the pyridine N and leads to a more potent leaving group. The chloro- and iodo-substituents at the 4-position of the pyridine were also found to be sensitive to a partial halogen exchange with bromide (entries 10 and 11).

Treatment of two equivalents of the corresponding aldehyde **12** with enantiopure (*R*, *R*)-1,2-diaminocyclohexane (**13**) in EtOH at room temperature for 15 h in the presence of 4 Å molecular sieves provided the salen ligands **14** (Scheme 3 and Table 1). The diimine formation from aldehydes **12** usually furnished **14** in high yields, with the exception of the 4-chloro- and the 4-cyanopyridine derivatives **14bH** and **14bI** (entries 11 and 12). In both cases none of the desired ligands was obtained, again arguably due to the pronounced leaving group properties of the more electron poor pyridines.

**Table 1:** Preparation of pyridinium aldehydes **12** and the ionic salen ligands **14**.

Entry	R^1^	R^2^	12	Yield 12 [%] ^a^	14	Yield 14 [%] ^a^
1	*t*Bu	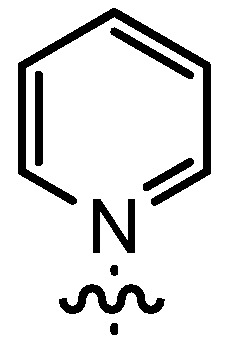	12aA	92	14aA	100
2	*n-*Pent	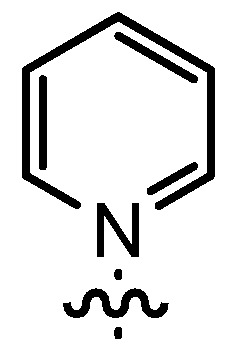	12bA	62	14bA	91
3	Me	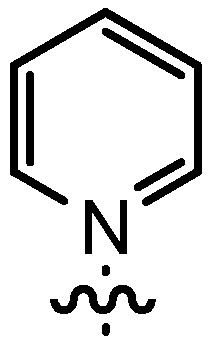	12cA	79	14cA	84
4	*t-*Bu	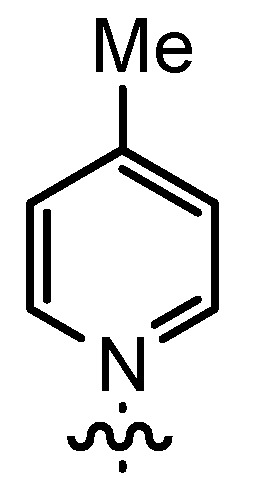	12aB	67	14aB	89
5	*n-*Pent	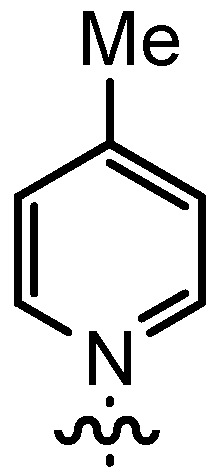	12bB	91	14bB	93
6	*n-*Pent	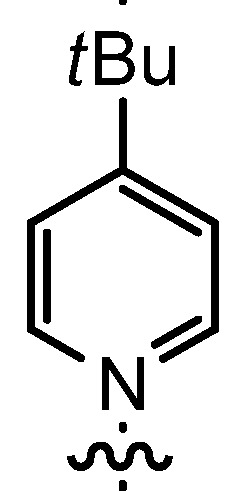	12bC	96	14bC	82
7	*n-*Pent	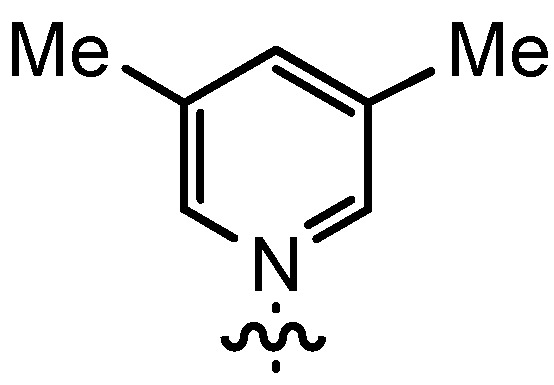	12bD	54	14bD	91
8	*n*Pent	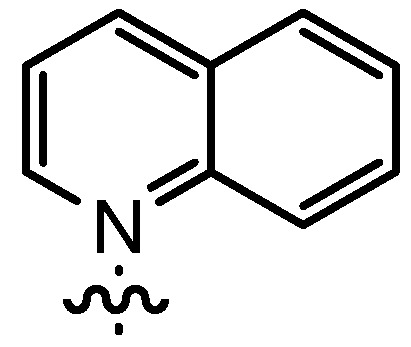	12bE	47	14bE	86
9	*n-*Pent	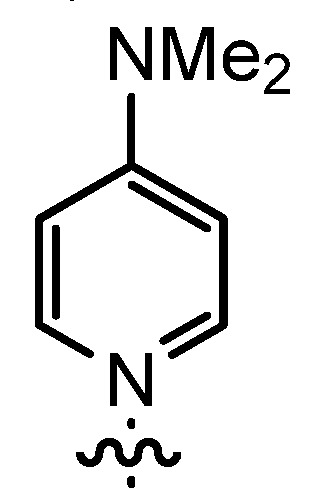	12bF	81	14bF	92
10	*n-*Pent	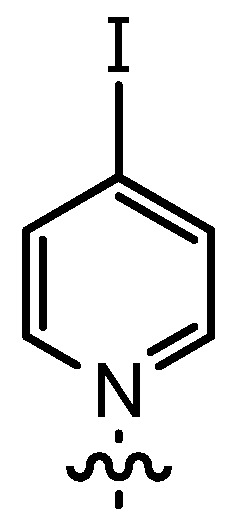	12bG	65	14bG	77
11	*n-*Pent	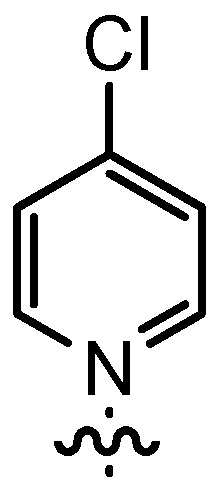	12bH	29	14bH	0
12	*n-*Pent	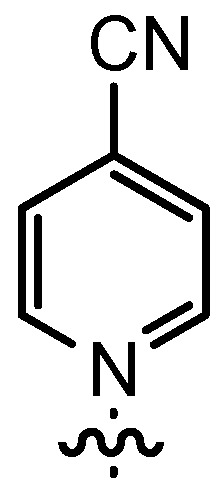	12bI	80	14bI	0

^a^ Yield of isolated product.

### 2.2. Catalysis

To examine the impact of the different pyridinium residues R^2^, all accessible ligands were investigated in the cyclocondensation of propionylbromide (**1A**) and dihydrocinnamaldehyde (**2a**, Table 2). Due to the air and moisture sensitivity of the investigated aluminum complexes **4**, the catalysts were generated *in situ* from 10 mol% of the corresponding ligand **14** and 10 mol% AlMe_3_ in CH_2_Cl_2_ at room temperature. We have previously already reported that the yields of β-lactone formation are generally higher with isolated catalysts, but isolation has only a small impact on enantio- and diastereoselectivity [53,54]. The catalyst solution was cooled to −70 °C and treated with both reagents and finally with *i*Pr_2_NEt. After stirring for 24 h at −70 °C, the reaction was terminated by addition of hydrochloric acid.

**Table 2:** Investigation of salen ligands **14** carrying different pyridinium moieties R^2^ in the cyclocondensation reaction of propionylbromide (**1A**) and dihydrocinnamaldehyde (**2a**) ^a^. 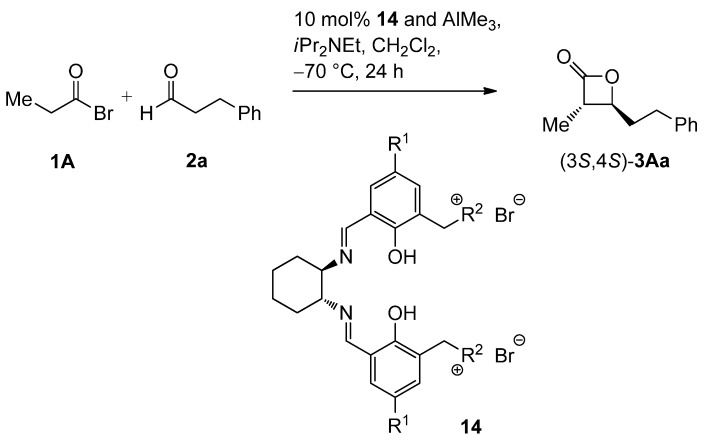

Entry	14	R^1^	R^2^	Conversion [%] ^b^	Yield 3Aa [%] ^b^	*dr* 3Aa [*trans*/*cis*] ^c^	*ee* 3Aa [%] ^d^
1	14aA	*t-*Bu	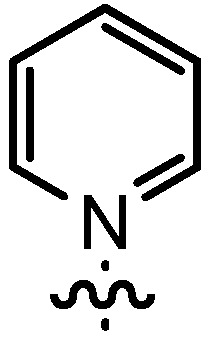	>95	91	93:7	89
2	14bA	*n-*Pent	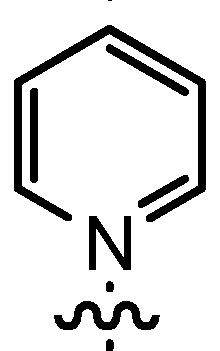	>98	98	93:7	88
3	14cA	Me	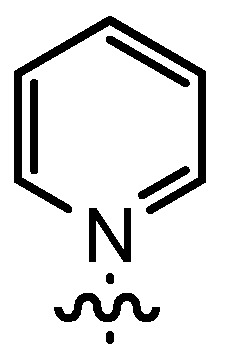	n.d.	71	91:9	88
4	14aB	*t-*Bu	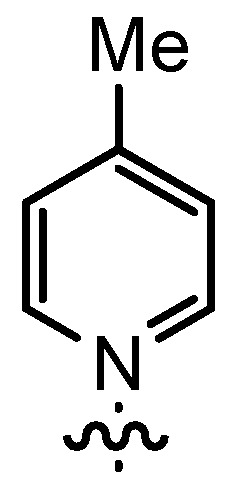	71	29	97:3	89
5	14bB	*n-*Pent	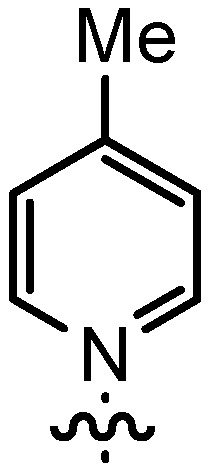	88	60	97:3	90
6	14bC	*n-*Pent	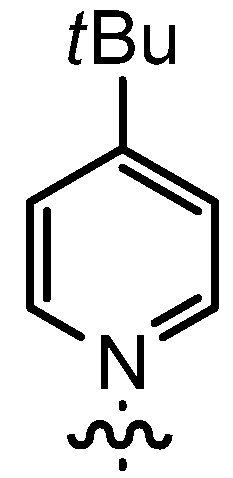	75	56	92:8	80
7	14bD	*n-*Pent	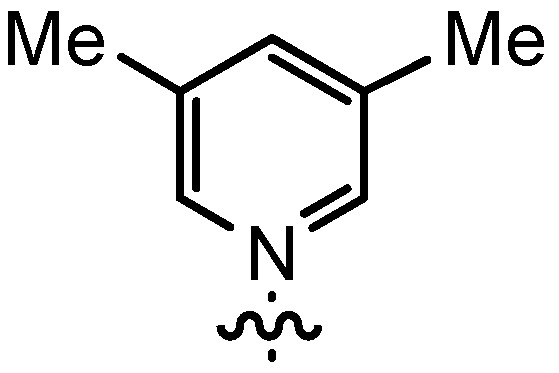	82	39	88:12	80
8	14bE	*n-*Pent	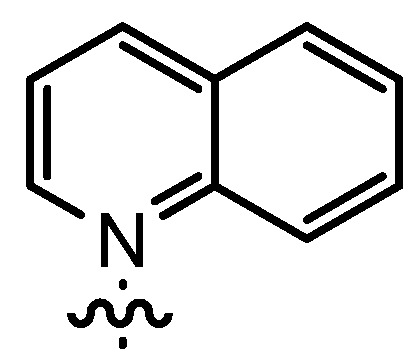	62	38	88:12	77
9	14bF	*n-*Pent	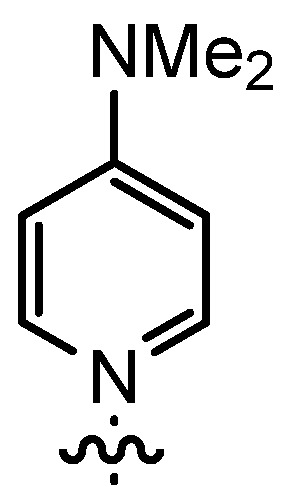	73	37	94:6	82
10	14bG	*n-*Pent	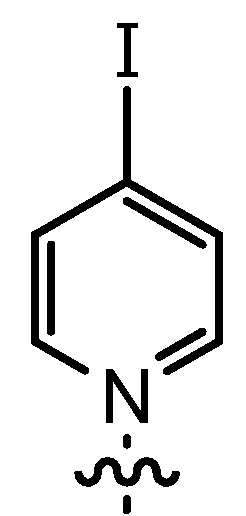	78	45	92:8	81

^a^ The catalyst was prepared *in situ* from **14** and AlMe_3_; ^b^ Determined by ^1^H-NMR using mesitylene as standard; ^c^ Determined by ^1^H-NMR; ^d^ Determined by HPLC on a chiral stationary phase.

In most cases, conversion of the aldehyde was good and yields of β-lactone **3Aa** were moderate to good. Catalysts generated from **14bA** and **14bB** with R^1^ = *n-*Pent (entries 2 and 5) resulted in higher conversions and yields than their direct counterparts derived from **14aA** and **14aB**, respectively, carrying a *t-*Bu residue R^1^ (entries 1 and 4). This is attributed to a higher solubility in CH_2_Cl_2_ at −70 °C with a more flexible alkyl chain [75]. **14cA** with R^1^ = Me resulted in poor solubility, explaining the lower yield (entry 3) compared to the results with **14aA** and **14bA**, respectively (entries 1 and 2). For that reason additional Me derivatives were not studied. 

Interestingly, the nature of R^1^ was found to have only a minor effect on both enantio- and diastereoselectivity (compare entries 1, 3, 4 and 5), whereas most salen catalyzed reactions require enhanced steric demand at that position for an efficient transfer of chirality [76]. In the present case, the stereoselectivity is much more dependent on the pyridine substituents. The best combination of enantioselectivity (*ee* = 90%) and diastereoselectivity (*trans*/*cis* = 97:3) was attained with ligand **14bB** carrying a 4-Me substituent as a weak σ-donor on the pyridine ring (entry 5). This ligand also allowed for a useful product yield. 

Increasing the steric demand of the pyridine by a 4-*t-*Bu substituent had only a minor impact on the reactivity, but both the *dr* (92:8) and the *ee* (80%) were negatively affected (entry 6). Reduced diastereoselectivity data were also noticed with a 3,5-dimethyl substitution pattern on the pyridine (entry 7) or with a quinoline residue (entry 8). 

The DMAP derivative **14bF** (entry 9) carrying the potent p-donor group NMe_2_ at the pyridine 4-position and the derivative **14bG** (entry 10) carrying an iodo atom as a weak σ-acceptor at the 4-position resulted in similar reactivity and stereoselectivity. For **14bF**, a lower diastereoselectivity was expected as a consequence of the +M-effect which should result in a wider charge distribution thereby weakening the postulated contact ion pair with the acyl halide enolate **6**. Consequently, σ-acceptors like in **14bG** should result in a more efficient ion pair formation and improved stereoselectivity, but steric effects as well as the poor solubility of **14bG** even at room temperature might have overwritten this effect. Unfortunately, it appears to be difficult to study the effect of σ-acceptors in ligands **14**, as they result in a lower stability of the catalysts.

In contrast, the 4-Me group does not only lead to an improved catalyst stability (the ligand **14bB** can be stored for at least two months at room temperature with no decomposition detected) and solubility, but might also have a positive effect on a uniform reactive conformation of the generated acyl halide enolate, whereas larger residues might hamper an efficient ion pair formation of the enolate and the pyridinium residue. 

The most stereoselective catalyst **4bB** was applied to different substrates (Table 3). For the investigated aldehydes and acyl bromides **4bB** gave always slightly higher enantioselectivities compared to our previously reported system **4aA**. 

**Table 3:** Asymmetric synthesis of *trans*-configured β-lactones **3** catalyzed by the aluminum bis-picolinium catalyst **4bB**. 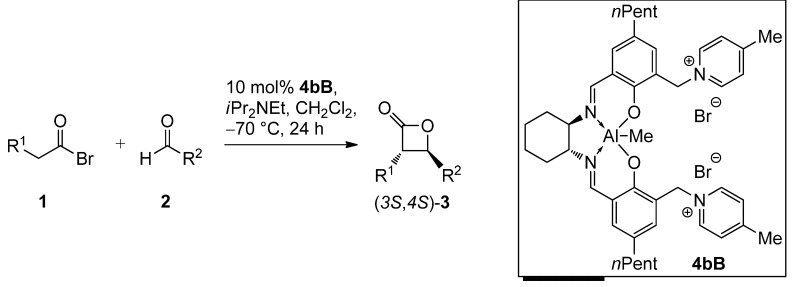

Entry	1	R^1^	2	R^2^	3	Yield [%] ^a^	*dr* [*trans*/*cis*] ^b^	*ee* [%] ^c^
1 ^d^	**1A**	Me	**2a**	(CH_2_)_2_Ph	**3Aa**	60	97:3 (97:3)	90 (88)
2 ^e^	**1A**	Me	**2a**	(CH_2_)_2_Ph	**3Aa**	69	97:3 (97:3)	90 (88)
3 ^d^	**1A**	Me	**2b**	Et	**3Ab**	59	98:2 (95:5)	90 (87)
4 ^d^	**1A**	Me	**2c**	Ph	**3Ac**	37	98:2 (94:6)	96 (91)
5 ^d^	**1B**	*n-*Pr	**2a**	(CH_2_)_2_Ph	**3Ba**	59	99:1 (98:2)	95 (94)

^a^ Yield of isolated product; ^b^ Diastereomeric ratio determined by ^1^H-NMR (data in brackets refer to the reported data obtained with 10 mol% catalyst **4aA**); ^c^ Determined by HPLC on a chiral stationary phase (data in brackets refer to the reported data obtained with 10 mol% catalyst **4aA**); ^d^ The catalyst was formed *in situ*; ^e^ The catalyst was isolated prior to use.

Moreover, the *trans*-selectivity was equal (entries 1 and 2) or better (entries 3 and 5) than with catalyst **4aA**. Entry 2 shows that improved yields can be obtained with the isolated catalyst **4bB**. However, the more convenient protocol with *in-situ* catalyst formation allows for similar diastereo- and enantioselectivity (compare entries 1 and 2). The highest enantioselectivity was attained with the aromatic benzaldehyde (entry 4, *ee* = 96%), but the yield is significantly lower than for aliphatic aldehydes. This is mainly a consequence of the marked sensitivity towards elimination of 4-aryl substituted 2-oxetanones explaining a partial decomposition during the workup [77].

### 2.3. Mechanistic and Theoretical Investigations

**Figure 3:**
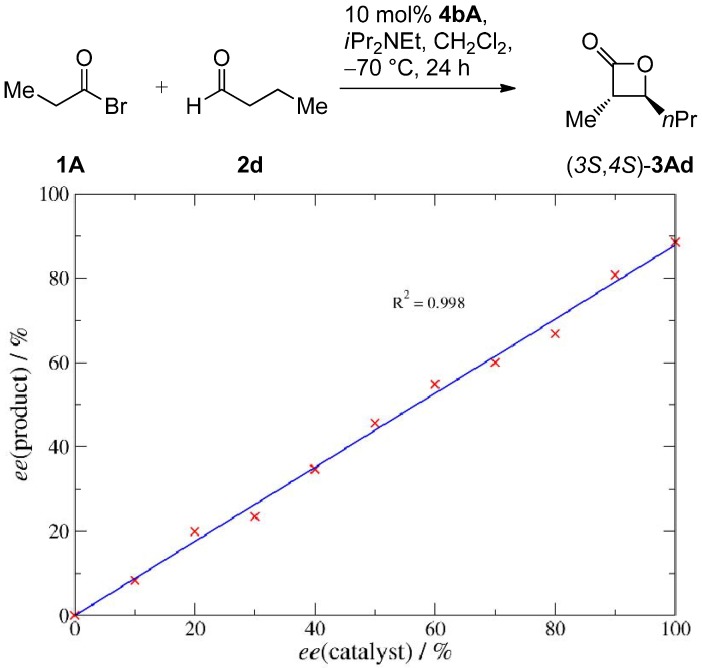
Investigation of a potential nonlinear effect for the formation of **3Ad** catalyzed by **4bA**.

Reactions catalyzed by salen complexes are known to proceed in several cases via bimetallic reaction pathways [78]. As part of our programme on bimetallic cooperative catalysis [79,80,81,82], we were therefore interested if two salen units might also cooperate to form the β-lactone products **3**. In that case the presence of a non-linear effect (NLE) would be expected [78,83]. The absence of a NLE in the present case (Figure 3) indicates that a major product formation pathway involving two salen molecules is most likely no realistic scenario. Our mechanistic considerations thus focus on reaction pathways involving a single catalyst molecule.

As mentioned above, we have previously shown that a cationic residue on the salen periphery is essential for high *trans*-selectivity, but also for high enantioselectivity and reactivity [53,54]. In the case of standard Al-salen complexes carrying a H atom or an isobutyl residue at the 3/3'-position, reactivity was poor and almost racemic product was formed favoring the *cis*-isomer. With a *t-*Bu residue no product was formed at all. In the initial study a pyridinium residue was found to be superior compared to ammonium residues [53,54]. We have tentatively explained this preference by a more efficient contact ion pair formation of the planar enolate moiety with the planar pyridinium system as compared to tetrahedral ammonium moieties.

As the positive charge of the substituent at the 3/3'-position is essential for a successful reaction outcome, we were interested in the effective charges for the derivatives described above. For that reason we accomplished a series of NBO analyses using the MOLPRO package of *ab initio* programmes [84]. For the electronic structure calculations we chose density functional theory in combination with a double-ζ basis set, *i.e.*, B3LYP/cc-pVDZ. Our original idea was that an acceptor substituent in the 4-position of the pyridinium ring might amplify the effect of the positive charge and could further stabilize the contact ion pair. In agreement with the literature [85], our calculations revealed a negative partial charge for the nitrogen atoms, for both tetramethylammonium and pyridinium cation systems (see Table 4). The positive partial charges in the tetramethylammonium reference are distributed over the nine hydrogen atoms. In contrast the pyridinium cation has positive partial charges on the α-carbon atoms, which might stabilize the enolate in the proposed contact ion pair more efficiently than in the case of quaternary ammonium cations, since the positive charge is wider distributed in the latter case. Pyridinium derivatives with substituents in the 4-position give nearly identical partial charges for the nitrogen and the α-carbon atoms as the parent system with a H atom in 4-position. Only the partial charge of the carbon in the 4-position shows a noticeable change on substitution. 

**Table 4:** Results of the NBO analysis.

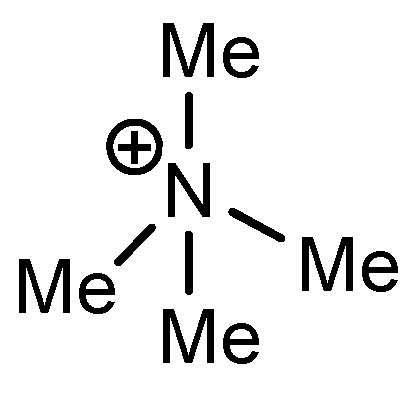		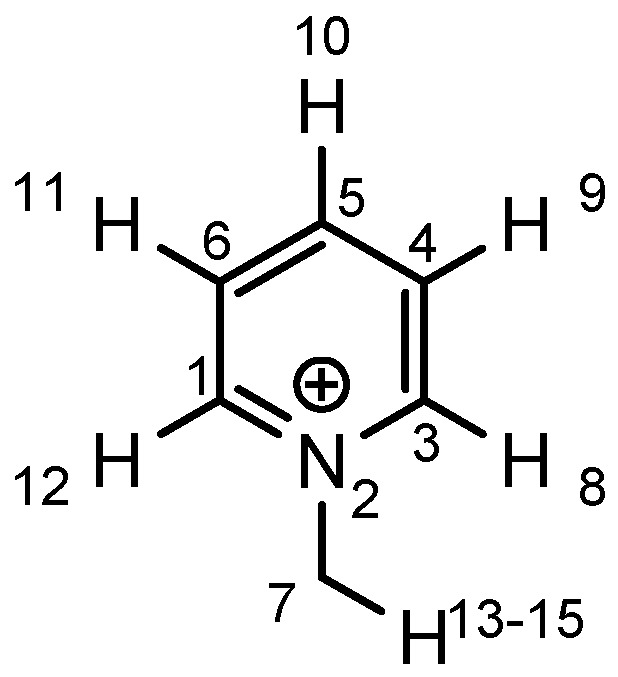		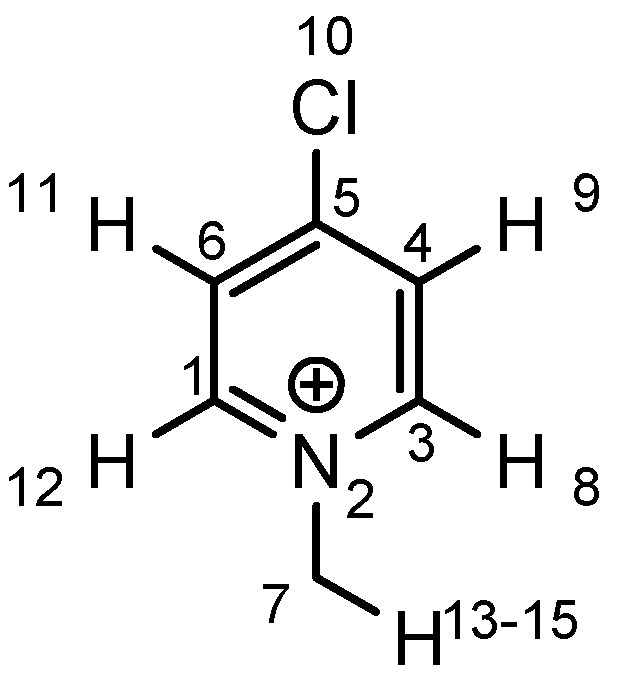		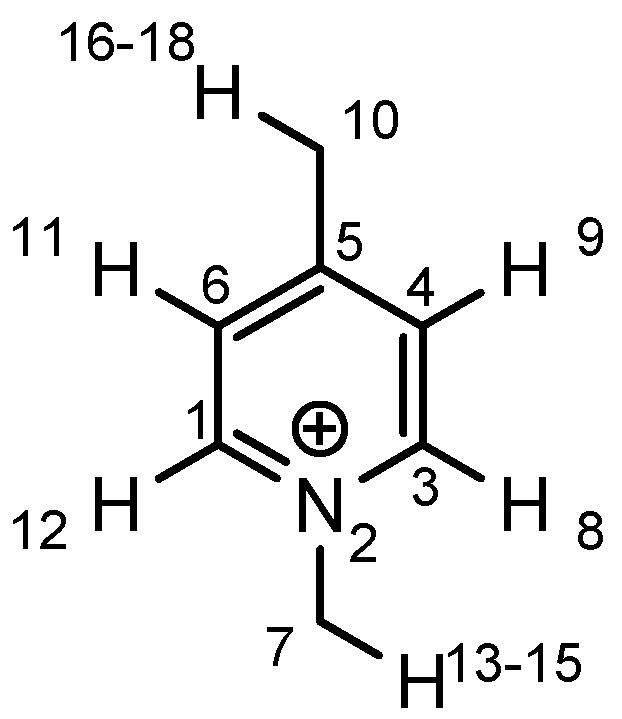	
**atom**	**charge**	**atom**	**charge**	**atom**	**charge**	**atom**	**charge**
N	−0.36	C ^(1)^	+0.11	C ^(1)^	+0.12	C ^(1)^	+0.11
C	−0.41	N ^(2)^	−0.35	N ^(2)^	−0.36	N ^(2)^	−0.36
H	+0.25	C ^(3)^	+0.11	C ^(3)^	+0.11	C ^(3)^	+0.10
		C ^(4)^	−0.22	C ^(4)^	−0.24	C ^(4)^	−0.22
		C ^(5)^	−0.12	C ^(5)^	+0.02	C ^(5)^	+0.07
		C ^(6)^	−0.22	C ^(6)^	−0.24	C ^(6)^	−0.23
		C ^(7)^	−0.41	C ^(7)^	−0.40	C ^(7)^	−0.41
		H ^(8)^	+0.26	H ^(8)^	+0.26	H ^(8)^	+0.26
		H ^(9)^	+0.28	H ^(9)^	+0.29	H ^(9)^	+0.27
		H ^(10)^	+0.27	Cl ^(10)^	+0.14	C ^(10)^	−0.67
		H ^(11)^	+0.28	H ^(11)^	+0.29	H ^(11)^	+0.27
		H ^(12)^	+0.26	H ^(12)^	+0.26	H ^(12)^	+0.26
		H ^(13–15)^	+0.25	H ^(13–15)^	+0.25	H ^(13–15)^	+0.25
						H ^(16–18)^	+0.26

Exemplarily, the 4-chloropyridinium cation is shown in Table 4 as an example of a σ-acceptor substituent and the 4-methylpyridinium cation bearing a σ-donor substituent. Hence the most important effects of the substituents are presumably: (1) the (de)stabilization of the bond between the pyridinium and the salen framework, since electron acceptors result in a higher leaving group tendency of the corresponding pyridine, whereas donor substituents have the opposite effect, and (2) the steric influence on the enolate.

**Scheme 4:**
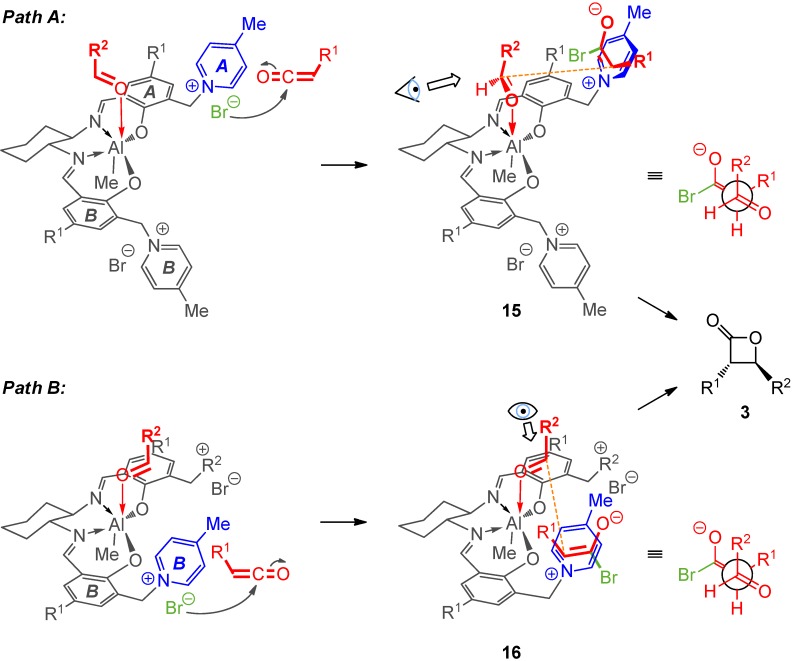
Two plausible reaction pathways via cooperative contact ion pair directed Lewis acid activation leading to the observed absolute and relative configuration of β-lactones **3**.

For the cooperative contact ion pair/Lewis acid activation two reaction pathways appear to be feasible (Scheme 4), both leading to the observed absolute and relative β-lactone configurations. The two reaction mechanisms differ in the reactive conformations of the coordinated aldehyde and in the pyridinium unit involved. In *path A* presented in Scheme 4, the aldehyde group’s H atom is expected to point toward the C=N imine bond in **15** connected to phenolate ring *A*. Pyridinium ring *A* forms the reactive contact ion pair with the acylbromoenolate. The depicted aldehyde conformation appears to be required to get the aldehyde in close distance to the reactive enolate in a transition state adopting the above proposed staggerd conformation (see Scheme 2) to form the *trans*-β-lactone with a (*3S*, *4S*)-configuration. 

In *path B* the same aldehyde H atom would point away from the chiral salen backbone. To form the product with the observed absolute configuration again the *Re*-face of the aldehyde has to react and this would require the action of the other pyridinium moiety *B*. 

We favor this scenario because the repulsive interactions of the aldehyde and the salen core would be minimized. An aldehyde conformation like in *path B* is often hampered in salen-complex catalyzed reactions by the presence of *t*Bu groups at the 3/3'-position [76,86]. However, the aldehyde conformation in *path A* appears more unfavorable in the present case given the folding of chiral salen metal complexes derived from *trans*-1,2-diaminocyclohexane which describes the ligand deviation from a planar arrangement [87,88,89]. In the present case phenolate ring *B* is expected, based on literature precedent for related Al salen complexes [90], to fold downward toward the Me-Al bond in a stepped conformation. That means, the *B*-half offers more available space for substrate accomodation.

## 3. Experimental

### 3.1. General

^1^H-NMR and ^13^C-NMR spectra were measured on a Bruker Avance spectrometer (300 or 500 MHz, Rheinstetten, Germany) in CDCl_3_ or DMSO as solvent using TMS as internal standard and chemical shifts are expressed as δ in ppm. Molecular masses were determined with the electron spray ionization (ESI) method on a MicroTOFQ (Bruker, Bremen, Germany) spectrometer. IR spectra were recorded on a Bruker Vector 22 FT-IR spectrometer (Bremen, Germany) with an ATR module (*Golden Gate*). Melting points are uncorrected and were measured on a Büchi Melting Point B-535 analysis device. The enantiomeric excesses were determined by HPLC on an Elite LaChrom system equipped with Hitachi modules. A chiral stationary phase Daicel column of the Chiracel OD-H type was used.

### 3.2. General Procedure for the Synthesis of ***10*** (GP1)

This procedure was according to a published protocol [72,73]. To a solution of the phenol derivative **9** (1 eq., 15.0 mmol) in acetonitrile, paraformaldehyde (6.7 eq.), magnesium chloride (1.5 eq.) and triethylamine (3.8 eq.) were added and the mixture was heated under reflux for 24 h. After cooling to ambient temperature, 1 M hydrochloric acid was added till the yellow residue dissolved, followed by extraction with diethyl ether (3 × 20 mL). The organic layers were dried over MgSO_4_, filtered and the solvent was removed *in vacuo*. Purification by column chromatography (petroleum ether/ethyl acetate 10:1) gave the aldehyde **10**.

*2-Hydroxy-5-tert-butylbenzaldehyde* (**10a**) was prepared according to GP1 using 4-*tert*-butylphenol (1 eq., 15.0 mmol, 2.25 g) and paraformaldehyde (6.7 eq., 101 mmol, 3.02 g). The product was formed as a yellow oil (13.5 mmol, 2.40 g, 90%). C_11_H_14_O_2_, MW: 178.23 g/mol. ^1^H-NMR (300 MHz, CDCl_3_, 20 °C): δ = 10.87 (*s*, 1H, Ar-O*H*), 9.89 (*s*, 1H, Ar-C*H*O), 7.58 (*dd*, *J* = 8.8, 2.5, 1H, *p-*Ar-*H*), 7.51 (*d*, *J* = 2.5, 1H, *o-*Ar-*H*), 6.94 (*d*, *J* = 8.7, 1H, *m*-Ar-*H*), 1.33 (*s*, 9H, Ar-C(C*H*_3_)_3_). The analytical data were in agreement with the literature values [72].

*2-Hydroxy-5-pentylbenzaldehyde* (**10b**) was prepared according to GP1 using 4-*n*-pentylphenol (1 eq., 8.0 mmol, 1.31 g, 1.4 mL) and paraformaldehyde (6.7 eq., 53.6 mmol, 1.61 g). The product was formed as a yellow oil (6.7 mmol, 1.29 g, 84%). C_12_H_16_O_2_, MW: 192.25 g/mol. ^1^H-NMR (300 MHz, CDCl_3_, 20 °C): δ = 10.85 (*s*, 1H, Ar-O*H*), 9.87 (*s*, 1H, Ar-C*H*O), 7.33–7.37 (*m*, 2H, *o-, p-*Ar-*H*), 6.91 (*d*, *J* = 8.5, 1H, *m*-Ar-*H*), 2.59 (*t*, *J* = 7.7, 2H, Ar-C*H*_2_(CH_2_)_3_CH_3_), 1.65–1.55 (*m*, 2H, Ar-CH_2_C*H*_2_(CH_2_)_2_CH_3_), 1.36–1.28 (*m*, 4H, Ar-(CH_2_)_2_(C*H*_2_)_2_CH_3_), 0.90 (*t*, *J* = 6.9, 3H, Ar-(CH_2_)_4_C*H*_3_). The analytical data are in agreement with the literature [91].

*2-Hydroxy-5-methylbenzaldehyde* (**10c**) was prepared according to GP1 using 4-methylphenol (1 eq., 14.0 mmol, 1.50 g) and paraformaldehyde (6.7 eq., 93.0 mmol, 2.80 g). The product was formed as a slightly yellow oil (10.5 mmol, 1.43 g, 76%) after Kugelrohr distillation (no column chromatography in this case). C_8_H_8_O_2_, MW: 136.15 g/mol. ^1^H-NMR (300 MHz, CDCl_3,_ 20 °C): δ = 10.84 (*s*, 1H, Ar-O*H*), 9.86 (*s*, 1H, Ar-C*H*O), 7.35–7.33 (*m*, 2H, *o*-, *p*-Ar-*H*), 6.90 (*d*, *J* = 9.2, 1H, *m*-Ar-*H*), 2.34 (s, 3H, C*H*_3_). The analytical data are in agreement with the literature [72].

### 3.3. General Procedure for the Synthesis of ***11*** (GP2)

This procedure was according to a published protocol [74]. To the corresponding aldehyde **10** (1 eq., 10.5 mmol) were added aq. hydrobromic acid (48%, 7.5 eq., 78.9 mmol, 8.6 mL), paraformaldehyde (1.5 eq., 15.8 mmol, 0.47 g) and a catalytic amount of sulfuric acid (3 drops). Depending on the alkyl chain R^1^, the mixture was stirred at 70 °C for 1 to 5 days. After cooling to ambient temperature, water (10 mL) was added followed by extraction with methylene chloride (3 × 10 mL). The collected organic layers were dried over Na_2_SO_4_, filtered and the solvent was removed *in vacuo*.

*3-(Bromomethyl)-2-hydroxy-5-tert-butylbenzaldehyde* (**11a**) was prepared according to GP2 using **10a** (1 eq., 13.5 mmol, 2.40 g), paraformaldehyde (1.5 eq., 20.2 mmol, 0.61 g) and aq. hydrobromic acid (48%, 7.5 eq., 101.1 mmol, 11.5 mL). After 1 day reaction time a brown oil (13.3 mmol, 3.61 g, 98%) was isolated. C_12_H_15_BrO_2_, MW: 271.15 g/mol. ^1^H-NMR (300 MHz, CDCl_3_, 20 °C): δ = 11.32 (*s*, 1H, Ar-O*H*), 9.90 (*s*, 1H, Ar-C*H*O), 7.64 (*d*, *J* = 2.5, 1H, Ar-*H*), 7.51 (*d*, *J* = 2.5, 1H, Ar-*H*), 4.59(*s*, 2H, CH_2_Br), 1.34 (*s*, 9H, C(CH_3_)_3_). The analytical data are in agreement with the literature [74].

*3-(Bromomethyl)-2-hydroxy-5-pentylbenzaldehyde* (**11b**) was prepared according to GP2 using **10b** (1 eq., 10.5 mmol, 2.02 g), paraformaldehyde (1.5 eq., 15.8 mmol, 0.47 g) and aq. hydrobromic acid (48%, 7.5 eq., 78.9 mmol, 8.6 mL). After 5 days reaction time a brown oil (9.77 mmol, 2.79 g, 93%) was isolated. C_13_H_17_BrO_2_, MW: 285.18 g/mol. M.p.: 32–34 °C. ^1^H-NMR (300 MHz, CDCl_3_, 20 °C): δ = 11.31 (*s*, 1H, Ar-O*H*), 9.87 (*s*, 1H, Ar-C*H*O), 7.43 (*d*, *J* = 2.1, 1H, Ar-*H*), 7.33 (*d*, *J* = 2.1, 1H, Ar-*H*), 4.57 (*s*, 2H, C*H*_2_Br), 2.59 (*t*, *J* = 7.8, 2H, Ar-C*H*_2_-C_4_H_9_), 1.61 (*m*, 2H, (C*H*_2_)_pentyl_), 1.33 (*m*, 4H, (C*H*_2_)_pentyl_), 0.90 (*t*, *J* = 6.9, 3H, CH_3_). ^13^C-NMR (75 MHz, CDCl_3_, 21 °C): *δ* = 196.5 (1C, *C*HO), 157.6 (1C, *C*_Ar_-OH), 138.3 (1C, *C*_Ar_-H), 134.5 (1C, *C*_Ar_-C_5_H_11_), 133.6 (1C, *C*_Ar_-H), 126.0 (1C, *C*_Ar_-CH_2_Br), 120.5 (1C, *C*_Ar_-CHO), 34.6 (1C, C_Ar_-*C*H_2_-C_4_H_9_), 31.3 (1C, (*C*H_2_)_pentyl_), 31.0 (1C, (*C*H_2_)_pentyl_), 26.8 (1C, C_Ar_-*C*H_2_Br), 22.5 (1C, (*C*H_2_)_pentyl_), 14.0 (1C, *C*H_3_). IR (solid): line graphic = 3046, 2954, 2930, 2854, 2753, 2185 (b), 1967 (b), 1811, 1646, 1438, 1209, 760. HRMS (EI)*m/z*: Anal. Calcd. for [C_13_H_17_BrO_2_^+^]: 284.0412, found 284.0412. 

*3-(Bromomethyl)-2-hydroxy-5-methylbenzaldehyde* (**11c**) was prepared according to GP2 using **10c** (1 eq., 9.0 mmol, 1.23 g), paraformaldehyde (1.5 eq., 13.5 mmol, 0.407 g) and aq. hydrobromic acid (48%, 7.5 eq., 67.5 mmol, 7.8 mL). After 70 h reaction time a beige-colored solid (5.8 mmol, 1.58 g, 76%) was isolated. C_9_H_9_BrO_2_, MW: 229.07 g/mol. ^1^H-NMR (300 MHz, CDCl_3,_ 20 °C): δ = 11.28 (*s*, 1H, Ar-O*H*), 9.84 (*s*, 1H, Ar-C*H*O), 7.42 (*d*, *J* = 1.9, 1H, *o*-Ar-*H*), 7.31 (*d*, *J* = 1.9, 1H, *p*-Ar-*H*), 4.54 (*s*, 2H, Ar-C*H*_2_-Br), 2.33 (s, 3H, C*H*_3_). The analytical data are in agreement with the literature [74].

### 3.4. General Procedure for the Synthesis of ***12*** (GP3)

This procedure was according to a published protocol [92]. To a solution of **11** (1 eq., 3.0 mmol) in acetonitrile (8 mL) was added the corresponding pyridine derivative (1.1 eq., 3.3 mmol). The mixture was stirred for 15 h at ambient temperature. For the workup the solvent was removed *in vacuo* till a volume of ca. 5 mL was reached and the product was precipitated with diethyl ether (10 mL). After drying *in vacuo* the salt **12** was obtained.

1*-(5-tert-Butyl-3-formyl-2-hydroxybenzyl)pyridinium bromide* (**12aA**) was prepared according to GP3 using **11a** (1 eq., 1.50 mmol, 406.7 mg) and pyridine (1.1 eq., 1.65 mmol, 130.5 mg, 133 µL). The product was formed as a white solid (1.39 mmol, 483.2 mg, 92%). C_17_H_20_BrNO_2_, MW: 350.25 g/mol. ^1^H-NMR (300 MHz, CDCl_3_, 20 °C): δ =11.37 (*s*, 1H, Ar-O*H*), 9.83 (*s*, 1H, Ar-C*H*O), 9.62 (*d*, *J* = 6.6, 2H, *o*-Py-*H*), 8.70 (*d*, *J* = 2.5, 1H, Ar-*H*), 8.38 (*tt*, *J* = 7.9, 1.3, 1H, *p*-Py-*H*), 7.97 (*t*, *J* = 7.0, 2H, *m*-Py-*H*), 7.57 (*d*, *J* = 2.5, 1H, Ar-*H*), 6.30 (*s*, 2H, Ar-C*H*_2_-Py), 1.32 (*s*, 9H, C(CH_3_)_3_). The analytical data are in agreement with the literature [54].

*1-(3-Formyl-2-hydroxy-5-pentylbenzyl)pyridinium bromide* (**12bA**) was prepared according to GP3 using **11b** (1 eq., 9.8 mmol, 2.79 g) and pyridine (1.1 eq., 10.7 mmol, 0.85 g, 866 µL). The product was formed as a beige-colored solid (6.1 mmol, 3.56 g, 62%). C_18_H_22_BrNO_2_, MW: 364.28 g/mol. M.p.: 146–147 °C. ^1^H-NMR (300 MHz, CDCl_3_, 20 °C): δ =14.45 (*s*, 1H, Ar-O*H*), 9.87 (*s*, 1H, Ar-C*H*O), 9.63 (*d*, *J* = 6.1, 2H, *o*-Py-*H*), 8.43 (*d*, *J* = 2.5, 1H, Ar-*H*), 8.39 (*tt*, *J* = 7.9, 1.3, 1H, *p*-Py-*H*), 7.99 (*t*, *J* = 7.2, 2H, *m*-Py-*H*), 7.47 (*d*, *J* = 2.1, 1H, Ar-*H*), 6.35 (*s*, 2H, Ar-C*H*_2_-Py), 2.67 (*t*, *J* = 8.0, 2H, Ar-C*H*_2_-C_4_H_9_), 1.64 (*m*, 2H, (C*H*_2_)_pentyl_), 1.33 (*m*, 4H, (C*H*_2_)_pentyl_), 0.89 (*t*, *J* = 6.9, 3H, C*H*_3_). ^13^C-NMR (75 MHz, CDCl_3_, 21 °C): *δ* = 196.6 (1C, *C*HO), 157.9 (1C, *C*_Ar_-OH), 145.6 (1C, α-*C*_Py_), 145.6 (1C, α-*C*_Py_), 145.1 (1C, γ-*C*_Py_), 140.2 (1C, *C*_Ar_-H), 136.2 (1C, *C*_Ar_-C_5_H_11_), 135.0 (1C, *C*_Ar_-CHO), 127.8 (2C, β-*C*_Py_), 121.1 (1C, *C*_Ar_-H), 120.6 (1C, *C*_Ar_-CH_2_-N_Py_), 59.2 (1C, C_Ar_-*C*H_2_-N_Py_), 34.5 (1C, C_Ar_-*C*H_2_-C_4_H_9_), 31.4 (1C, (*C*H_2_)_Pentyl_). 31.0 (1C, (*C*H_2_)_Pentyl_), 22.5 (1C, (*C*H_2_)_Pentyl_), 14.1 (1C, *C*H_3_). IR (solid): line graphic = 3046, 2949, 2860, 2196, 1966, 1641,1627, 1154, 690. HRMS (ESI) *m/z*: Anal. Calcd. for [[C_18_H_22_NO_2_]^+^]: 284.1645, found 284.1641.

*1-(3-Formyl-2-hydroxy-5-methylbenzyl)pyridinium bromide* (**12cA**) was prepared according to GP3 using **11c** (1 eq., 7.0 mmol, 1.62 g) and pyridine (1.1 eq., 7.7 mmol, 597 μL). The product was formed as a colorless solid (6.5 mmol, 1.99 g, 92%). C_14_H_14_BrNO_2_, MW: 308.17 g/mol. M.p.: 189–192 °C. ^1^H-NMR (300 MHz, CDCl_3_, 20 °C): δ = 11.39 (*s*, 1H, Ar-O*H*), 9.84 (*s*, 1H, Ar-C*H*O), 9.63 (*d*, *J* = 5.7, 2H, *o*-Py-*H*), 8.44 (*m*, 2H, *p*-C_Py_-*H* und C_Ar_-*H*), 8.02 (*t*, *J* = 7.2, 2H, *m*-Py-*H*), 7.44 (*d*, *J* = 1.9, 1H, *o*-Ar-*H*), 6.33 (*s*, 2H, Ar-C*H*_2_-Py), 2.39 (*s*, 9H, C*H*_3_).^ 13^C-NMR (75 MHz, CDCl_3,_ 20 °C): δ = 196.6 (CO), 157.7 (*C*_Ar_-OH), 145.6 (α-Py-*C*), 145.1 (α-Py-*C*), 140.8 (γ-Py-*C*), 135.7 (*C*_Ar_H), 135.7 (*C*_Ar_-CH_3_), 131.0 (*C*_Ar_-CO), 127.8 (2 x β-Py-*C*), 121.1 (*C*_Ar_-CH_2_-Py), 120.5 (*C*_Ar_H), 58.9 (C_Ar_-*C*H_2_-Py), 20.2 (*C*H_3_). IR (solid): line graphic = 3446, 3378, 3117, 3046, 2927, 2051, 1882, 1626, 1463. MS (ESI) *m/z*: 228.1 (20%, M^+^), 149.1 (100%, M^+^–pyridine), 133.1 (10%, M^+^–pyridine–OH).

*1-(5-tert-Butyl-3-formyl-2-hydroxybenzyl)-4-methylpyridinium bromide* (**12aB**) was prepared according to GP3 using **11a** (1 eq., 3.00 mmol, 813.5 mg) and picoline (1.1 eq., 3.30 mmol, 307.3 mg, 321 µL). The product was formed as a white solid (2.02 mmol, 734.9 mg, 67%). C_18_H_22_BrNO_2_, MW: 364.28 g/mol. M.p.: 247.2–248.0 °C. ^1^H-NMR (300 MHz, DMSO, 20 °C): δ = 11.16 (*b*, 1H, Ar-O*H*), 10.06 (*s*, 1H, Ar-C*H*O), 8.99 (*d*, *J* = 6.7, 2H, *o*-Py-*H*), 8.09 (*d*, *J* = 2.4, 1H, Ar-*H*), 7.98 (*d*, *J* = 6.5, 2H, *m*-Py-*H*), 7.89 (*d*, *J* = 2.6, 1H, Ar-*H*), 5.82 (*s*, 2H, Ar-C*H*_2_-Py), 2.59 (*s*, 3H, Py-C*H*_3_), 1.32 (*s*, 9H, C(C*H*_3_)_3_). ^13^C-NMR (125 MHz, DMSO, 20 °C): δ = 196.5, 159.3, 156.7, 143.9, 142.8, 136.1, 130.8, 128.3, 122.0, 121.2, 58.2, 34.1, 31.0, 21.4. IR (solid): line graphic = 3419, 3018, 2951, 2864, 1639, 1473, 1379, 1280, 1226, 1151, 1018, 826, 761, 700, 617. MS (ESI) *m/z*: 284.2 (57%, [M]^+^), 191.1 (100%, [M]^+^−[Py]), 94.1 (15%, [PyH]^+^). HRMS (ESI) *m/z*: Anal. Calcd. for [[C_18_H_22_NO_2_]^+^]: 284.1645, found: 284.1634.

*1-(3-Formyl-2-hydroxy-5-pentylbenzyl)-4-methylpyridinium bromide* (**12bB**) was prepared according to GP3 using **11b** (1 eq., 14.0 mmol, 4.0 g) and picoline (1.1 eq., 15.4 mmol, 1.4 g, 1.5 mL). The product was formed as a yellow solid (12.8 mmol, 4.8 g, 91%). C_19_H_24_BrNO_2_, MW: 378.30 g/mol. M.p.: 118.2–120.6 °C. ^1^H-NMR (300 MHz, CDCl_3_, 20 °C): δ = 11.41 (*s*, 1H, Ar-O*H*), 9.86 (*s*, 1H, Ar-C*H*O), 9.40 (*d*, *J* = 6.7, 2H, *o*-Py-*H*), 8.39 (*d*, *J* = 2.0, 1H, Ar-*H*), 7.72 (*d*, *J* = 6.4, 2H, *m*-Py-*H*), 7.45 (*d*, *J* = 2.1, 1H, Ar-*H*), 6.24 (*s*, 2H, Ar-C*H*_2_-Py), 2.68–2.63 (*m*, 5H, Ar-C*H*_2_-C_4_H_9_ and Py-C*H*_3_), 1.68–1.58 (*m*, 2H, (C*H*_2_)_pentyl_), 1.38–1.25 (*m*, 4H, (C*H*_2_)_pentyl_), 0.89 (*t*, *J* = 6.8, 3H, C*H*_3_). ^13^C-NMR (75 MHz, CDCl_3_, 20 °C): δ = 196.6, 158.9, 157.9, 144.6, 140.3, 136.2, 134.9, 128.2, 121.3, 120.5, 58.3, 34.5, 31.4, 31.0, 22.5, 22.3, 14.1. IR (solid): line graphic = 3013, 2927, 2855, 1637, 1466, 1278, 1151, 1016, 830, 748, 699. MS (ESI) *m/z*: 298.2 (100%, [M]^+^), 205.1 (13%, [M]^+^−[Py]). HRMS (ESI) *m/z*: Anal. Calcd. for [[C_19_H_24_NO_2_]^+^]: 298.1802, found: 298.1793.

*4-tert-Butyl-1-(3-formyl-2-hydroxy-5-pentylbenzyl)-pyridinium bromide* (**12bC**) was prepared according to GP3 using **11b** (1 eq., 1.0 mmol, 285.2 mg) and 4-*tert*-butylpyridine (1.1 eq., 1.1 mmol, 148.7 mg, 107 µL). The product was formed as a yellow solid (0.96 mmol, 403.4 mg, 96%). C_22_H_30_BrNO_2_, MW: 420.38 g/mol. M.p.: 188.6–189.8 °C. ^1^H-NMR (300 MHz, CDCl_3_, 20 °C): δ = 11.45 (*s*, 1H, Ar-O*H*), 9.87 (*s*, 1H, Ar-C*H*O), 9.50 (*d*, *J* = 6.8, 2H, *o*-Py-*H*), 8.46 (*d*, *J* = 2.0, 1H, Ar-*H*), 7.86 (*d*, *J* = 6.8, 2H, *m*-Py-*H*), 7.45 (*d*, *J* = 2.0, 1H, Ar-*H*), 6.24 (*s*, 2H, Ar-C*H*_2_-Py), 2.67 (*t*, *J* = 7.7, 2H, Ar-C*H*_2_-C_4_H_9_), 1.69–1.59 (*m*, 2H, (C*H*_2_)_pentyl_), 1.39 (*s*, 9H, Py-C(C*H*_3_)_3_). 1.37–1.25 (*m*, 4H, (C*H*_2_)_pentyl_), 0.89 (*t*, *J* = 6.8, 3H, C*H*_3_). ^13^C-NMR (75 MHz, CDCl_3_, 20 °C): δ = 196.7, 157.9, 144.8, 140.5, 136.3, 134.8, 124.7, 121.3, 120.5, 58.0, 36.6, 34.5, 31.4, 31.0, 30.0, 22.5, 14.1. IR (solid): line graphic = 3035, 2956, 2928, 2857, 1643, 1462, 1384, 1270, 1219, 1169, 1109, 1015, 849, 749, 712, 647, 561. MS (ESI) *m/z*: 340.2 (15%, [M]^+^), 205.1 (100%, [M]^+^−[Py]). HRMS (ESI) *m/z*: Anal. Calcd. for [[C_22_H_30_NO_2_]^+^]: 340.2271, found: 340.2255.

*3*,*5-Dimethyl-1-(3-formyl-2-hydroxy-5-pentylbenzyl)-pyridinium bromide* (**12bD**) was prepared according to GP3 using **11b** (1 eq., 1.0 mmol, 285.2 mg) and 3,5-dimethylpyridine (1.1 eq., 1.1 mmol, 117.9 mg, 126 µL). The product was formed as a yellow solid (0.54 mmol, 211.4 mg, 54%). C_20_H_26_BrNO_2_, MW: 392.33 g/mol. M.p.: 150.3–151.3 °C. ^1^H-NMR (300 MHz, CDCl_3_, 20 °C): δ = 11.37 (*s*, 1H, Ar-O*H*), 9.86 (*s*, 1H, Ar-C*H*O), 9.24 (*b*, 2H, *o*-Py-*H*), 8.39 (*b*, 1H, Ar-*H*), 7.94 (*s*, 1H, *p*-Py-*H*), 7.44 (*m*, 1H, Ar-*H*), 6.15 (*s*, 2H, Ar-C*H*_2_-Py), 2.64 (*t*, *J* = 7.8, 2H, Ar-C*H*_2_-C_4_H_9_), 2.54 (*s*, 6H, Py-C*H*_3_). 1.61 (*m*, 2H, (C*H*_2_)_pentyl_), 1.30 (*m*, 4H, (C*H*_2_)_pentyl_), 0.86 (*t*, *J* = 6.6, 3H, C*H*_3_). ^13^C-NMR (125 MHz, CDCl_3_, 20 °C): δ = 196.7, 157.9, 146.2, 142.3, 140.5, 138.5, 136.1, 134.8, 121.3, 120.6, 58.5, 34.5, 31.4, 30.9, 22.5, 18.6, 15.3, 14.1. IR (solid): line graphic = 2994, 2927, 2869, 1650, 1467, 1279, 1224, 1020, 1005, 922, 871, 781, 738, 699. MS (ESI) *m/z*: 312.2 (34%, [M]^+^), 205.1 (100%, [M]^+^−[Py]), 108.1 (52%, [PyH]^+^). HRMS (ESI) *m/z*: Anal. Calcd. for [[C_20_H_26_NO_2_]^+^]: 312.1958, found: 312.1962.

*1-(3-Formyl-2-hydroxy-5-pentylbenzyl)-quinolinium bromide* (**12bE**) was prepared according to GP3 using **11b** (1 eq., 1.0 mmol, 285.2 mg) and quinoline (1.1 eq., 1.1 mmol, 142.1 mg, 129 µL). The product was formed as a white solid (0.47 mmol, 194.0 mg, 47%). C_22_H_24_BrNO_2_, MW: 414.34 g/mol. M.p.: 215.1–216.4 °C. ^1^H-NMR (300 MHz, DMSO, 20 °C): δ = 11.20 (*s*, 1H, Ar-O*H*), 10.04 (*s*, 1H, Ar-C*H*O), 9.68 (*d*, *J* = 5.8, 1H, quinoline-*H*), 9.37 (*d*, *J* = 8.3, 1H, quinoline-*H*), 8.58–8.50 (*m*, 2H, quinoline-*H*), 8.29–8.19 (*m*, 2H, quinoline-*H*), 8.03 (*t*, *J* = 7.6, 1H, quinoline-*H*), 7.65 (*d*, *J* = 1.8, 1H, Ar-*H*), 7.57 (*d*, *J* = 1.8, 1H, Ar-*H*), 6.32 (*s*, 2H, Ar-C*H*_2_-quinoline), 2.52 (*m*, 2H, Ar-C*H*_2_-C_4_H_9_), 1.50 (*m*, 2H, (C*H*_2_)_pentyl_), 1.20 (*m*, 4H, (C*H*_2_)_pentyl_), 0.80 (*t*, *J* = 6.9, 3H, C*H*_3_). ^13^C-NMR (125 MHz, DMSO, 20 °C): δ = 196.2, 156.3, 150.7, 148.1, 137.6, 137.0, 135.7, 134.3, 133.1, 130.9, 129.9, 129.7, 122.1, 121.7, 121.3, 118.9, 55.7, 33.6, 30.5, 30.3, 21.8, 13.9. IR (solid): line graphic = 3024, 2924, 2852, 1644, 1620, 1527, 1449, 1370, 1278, 1224, 1158, 1012, 821, 785, 709. MS (EI) *m/z*: 334.2 (32%, [M]^+^), 205.1 (100%, [M]^+^−[quinoline]). HRMS (ESI) *m/z*: Anal. Calcd. for [[C_22_H_24_NO_2_]^+^]: 334.1802, found: 334.1796.

*4-Dimethylamino-1-(3-formyl-2-hydroxy-5-pentylbenzyl)-pyridinium bromide* (**12bF**) was prepared according to GP3 using **11b** (1 eq., 1.0 mmol, 285.2 mg) and 4-dimethylaminopyridine (1.1 eq., 1.1 mmol, 134.4 mg). The product was formed as a beige-colored solid (0.81 mmol, 330.2 mg, 81%). C_20_H_27_BrN_2_O_2_, MW: 407.34 g/mol. M.p.: 187.6–188.9 °C. ^1^H-NMR (300 MHz, CDCl_3_, 20 °C): δ = 11.29 (*b*, 1H, Ar-O*H*), 9.85 (*s*, 1H, Ar-C*H*O), 8.68 (*d*, *J* = 7.8, 2H, *o*-Py-*H*), 8.08 (*d*, *J* = 2.2, 1H, Ar-*H*), 7.38 (*d*, *J* = 2.2, 1H, Ar-*H*), 6.88 (*d*, *J* = 7.8, 2H, *m*-Py-*H*), 5.65 (*s*, 2H, Ar-C*H*_2_-Py), 3.22 (*s*, 6H, Py-C*H*_3_), 2.61 (*t*, *J* = 7.8, 2H, Ar-C*H*_2_-C_4_H_9_), 1.60 (*m*, 2H, (C*H*_2_)_pentyl_), 1.29 (*m*, 4H, (C*H*_2_)_pentyl_), 0.86 (*t*, *J* = 6.8, 3H, C*H*_3_). ^13^C-NMR (75 MHz, CDCl_3_, 20 °C): δ = 196.6, 157.8, 156.2, 143.0, 139.7, 135.7, 134.2, 122.4, 120.5, 107.8, 55.3, 40.4, 34.5, 31.4, 31.0, 22.5, 14.1. IR (solid): line graphic = 3065, 2955, 2927, 2855, 1640, 1562, 1443, 1402, 1384, 1261, 1164, 1013, 943, 838, 820, 770, 715, 702. MS (EI) *m/z*: 327.2 (68%, [M]^+^), 205.1 (70%, [M]^+^−[Py]), 123.1 (100%, [PyH]^+^). HRMS (ESI) *m/z*: Anal. Calcd. for [[C_20_H_27_N_2_O_2_]^+^]: 327.2067, found: 327.2069.

*1-(3-Formyl-2-hydroxy-5-pentylbenzyl)-4-iodopyridinium bromide* (**12bG**) was prepared according to GP3 using **11b** (1 eq., 1.0 mmol, 285.2 mg) and 4-iodopyridine (1.1 eq., 1.1 mmol, 225.5 mg). The product was formed as a green solid (0.68 mmol, 332.7 mg, 65%). C_18_H_21_BrINO_2_, MW: 490.17 g/mol. M.p.: 182.5–183.3 °C (decomposition). ^1^H-NMR (300 MHz, CDCl_3_, 20 °C): δ = 11.34 (*s*, 1H, Ar-O*H*), 9.86 (*s*, 1H, Ar-C*H*O), 9.21 (*d*, *J* = 6.7, 2H, *o*-Py-*H*), 8.35 (*d*, *J* = 2.0, 1H, Ar-*H*), 8.30 (*d*, *J* = 6.8, 2H, *m*-Py-*H*), 7.47 (*d*, *J* = 2.1, 1H, Ar-*H*), 6.22 (*s*, 2H, Ar-C*H*_2_-Py), 2.66 (*t*, *J* = 7.7, 2H, Ar-C*H*_2_-C_4_H_9_), 1.69–1.58 (*m*, 2H, (C*H*_2_)_pentyl_), 1.38–1.28 (*m*, 4H, (C*H*_2_)_pentyl_), 0.89 (*t*, *J* = 6.8, 3H, C*H*_3_). ^13^C-NMR (125 MHz, CDCl_3_, 20 °C): δ = 196.0, 156.7, 144.1, 138.3, 137.1, 134.2, 133.4, 121.8, 121.5, 120.9, 58.5, 33.6, 30.7, 30.2, 21.8, 13.8. IR (solid): line graphic = 3012, 2959, 2928, 2856, 1645, 1615, 1448, 1275, 1261, 1223, 1153, 1017, 837, 807, 703. MS (ESI) *m/z*: 410.1 (9%, [M]^+^), 205.1 (100%, [M]^+^−[Py]). HRMS (ESI) *m/z*: Anal. Calcd. for [[C_18_H_21_INO_2_]^+^]: 410.0611, found: 410.0609.

*4-Chloro-1-(3-formyl-2-hydroxy-5-pentylbenzyl)-pyridinium bromide* (**12bH**) was prepared according to GP3 using **11b** (1 eq., 1.0 mmol, 285.2 mg) and 4-chloropyridine (1.1 eq., 1.1 mmol, 187.3 mg, 156 µL). The product was formed as a beige-colored solid (0.67 mmol, 267.2 mg, 29%). C_18_H_21_BrClNO_2_, MW: 398.72 g/mol. M.p.: 131.6–133.2 °C. ^1^H-NMR (500 MHz, CDCl_3_, 20 °C): δ = 11.35 (*s*, 1H, Ar-O*H*), 9.84 (*s*, 1H, Ar-C*H*O), 9.59 (*d*, *J* = 6.8, 2H, *o*-Py-*H*), 8.29 (*m*, 1H, Ar-*H*), 7.94 (*d*, *J* = 6.8, 2H, *m*-Py-*H*), 7.44 (*m*, 1H, Ar-*H*), 6.31 (*s*, 2H, Ar-C*H*_2_-Py), 2.63 (*t*, *J* = 7.8, 2H, Ar-C*H*_2_-C_4_H_9_), 1.60 (*m*, 2H, (C*H*_2_)_pentyl_), 1.30 (*m*, 4H, (C*H*_2_)_pentyl_), 0.86 (*t*, *J* = 6.6, 3H, C*H*_3_). ^13^C-NMR (125 MHz, CDCl_3_, 20 °C): δ = 196.5, 157.9, 154.0, 146.6, 139.9, 136.1, 135.0, 128.1, 121.0, 120.6, 59.0, 34.5, 31.4, 31.0, 22.5, 14.1. IR (solid): line graphic = 3021, 2952, 2925, 2857, 1650, 1622, 1493, 1456, 1275, 1224, 1212, 1108, 1020, 845, 821, 713, 697. MS (EI) *m/z*: 318.1 (100%, [M]^+^), 205.1 (54%, [M]^+^−[Py]). HRMS (ESI) *m/z*: Anal. Calcd. for [[C_18_H_21_ClNO_2_]^+^]: 318.1255, found: 318.1255.

*4-Cyano-1-(3-formyl-2-hydroxy-5-pentylbenzyl)-pyridinium bromide* (**12bI**) was prepared according to GP3 using **11b** (1 eq., 1.0 mmol, 285.2 mg) and 4-cyanopyridine (1.1 eq., 1.1 mmol, 114.5 mg). The product was formed as a yellow solid (0.80 mmol, 312.5 mg, 80%). C_19_H_21_BrN_2_O_2_, MW: 389.29 g/mol. M.p.: 131.6–133.2 °C. ^1^H-NMR (300 MHz, CDCl_3_, 20 °C): δ = 11.43 (*s*, 1H, Ar-O*H*), 9.88–.85 (*m*, 3H, Ar-C*H*O and *o*-Py-*H*), 8.30 (*m*, 1H, Ar-*H*), 8.23 (*d*, *J* = 6.5, 2H, *m*-Py-*H*), 7.49 (*m*, 1H, Ar-*H*), 6.46 (*s*, 2H, Ar-C*H*_2_-Py), 2.66 (*t*, *J* = 7.8, 2H, Ar-C*H*_2_-C_4_H_9_), 1.63 (*m*, 2H, (C*H*_2_)_pentyl_), 1.33 (*m*, 4H, (C*H*_2_)_pentyl_), 0.89 (*t*, *J* = 6.8, 3H, C*H*_3_). ^13^C-NMR (125 MHz, CDCl_3_, 20 °C): δ = 196.5, 157.9, 147.3, 139.9, 136.2, 135.3, 130.1, 128.1, 120.7, 120.4, 113.7, 60.8, 34.5, 31.4, 31.0, 22.5, 14.1. IR (solid): line graphic = 3112, 2960, 2928, 2849, 1654, 1635, 1603, 1456, 1274, 1189, 1142, 1010, 830, 716, 557. MS (EI) *m/z*: 309.2 (11%, [M]^+^), 205.1 (100%, [M]^+^−[Py]). HRMS (ESI) *m/z*: Anal. Calcd. for [[C_19_H_21_N_2_O_2_]^+^]: 309.1598, found: 309.1606.

### 3.5. General Procedure for the Synthesis of ***14*** (GP4)

This procedure is according to a published protocol [54]. To a solution of (1*R*,2*R*)-(−)-1,2-diaminocyclohexane (1 eq., 0.15 mmol, 17.1 mg) in ethanol (0.7 mL) at ambient temperature molecular sieves (4 Å) and the corresponding salt **12** (2 eq., 0.30 mmol) were added. The mixture was stirred for 15 h at ambient temperature. For workup the mixture was filtered and washed with ethanol. The solvent was removed*in vacuo* till a volume of 5 mL was reached and the ligand was precipitated with diethyl ether (30 mL). The solid product was then dried *in vacuo*.

*(R*,*R)-(−)-N*,*N'-Bis(3-tert-butyl-5-(pyridinium-1-ylmethyl)salicylidene)-1*,*2-cyclohexanediamine dibromide* (**14aA**) was prepared according to GP4 using **12aA** (2 eq., 1.06 mmol, 371.3 mg) and (1*R*,2*R*)-(−)-1,2-diaminocyclohexane (1 eq., 0.53 mmol, 60.5 mg). The product was formed as an orange solid (0.53 mmol, 438.0 mg, 100%). C_40_H_50_Br_2_N_4_O_2_, MW: 778.66 g/mol. ^1^H-NMR (300 MHz, CD_2_Cl_2_, 20 °C): δ = 14.16 (*b*, 2H, Ar-O*H*), 9.55 (*d*, *J* = 5.5, 4H, *o*-Py-*H*), 8.46 (*t*, *J* = 7.8, 2H, *p*-Py-*H*), 8.38 (*s*, 2H, Ar-C*H*=N-Cy), 8.22 (*d*, *J* = 2.5, 2H, Ar-*H*), 7.99 (*m*, 4H, *m*-Py-*H*), 7.32 (*d*, *J* = 2.5, 2H, Ar-*H*), 6.13 (*m*, 4H, Ar-C*H*_2_-Py), 3.43 (*d*, *J* = 9.5, 2H, Cy-*H*N=CHR)), 1.90 (*m*, 4H, Cy-*H*_2_), 1.67 (*m*, 2H, Cy-*H*_2_), 1.47 (*m*, 2H, Cy-*H*_2_), 1.27 (*s*, 18H, C(CH_3_)_3_). The analytical data are in agreement with the literature [54].

*(R*,*R)-(−)-N*,*N'-Bis(3-pentyl-5-(pyridinium-1-ylmethyl)salicylidene)-1*,*2-cyclohexanediamine dibromide* (**14bA**) was prepared according to GP4 using **12bA** (2 eq., 0.90 mmol, 328.0 mg) and (1*R*,2*R*)-(−)-1,2-diaminocyclohexane (1 eq., 0.45 mmol, 51.4 mg). The product was formed as an orange solid (0.41 mmol, 329.3 mg, 91%). C_42_H_54_Br_2_N_4_O_2_, MW: 806.71 g/mol. M.p.: 128.4–129.6 °C. line graphic (*c* = 10 mg/mL, CH_2_Cl_2_) = −273.0. ^1^H-NMR (300 MHz, CD_2_Cl_2_, 20 °C): δ = 14.13 (*b*, 2H, Ar-O*H*), 9.44 (*d*, *J* = 9.7, 4H, *o*-Py-*H*), 8.41 (*tt*, *J* = 7.9, 1.4, 2H, *p*-Py-*H*), 8.32 (*s*, 2H, Ar-C*H*=N-Cy), 7.94 (*t*, *J* = 7.2, 4H, *m*-Py-*H*), 7.81 (*d*, *J* = 2.2, 2H, Ar-*H*), 7.13 (*d*, *J* = 1.9, 2H, Ar-*H*), 6.09 (*m*, 4H, Ar-C*H*_2_-Py), 3.41 (*m*, 2H, Cy-*H*N=CHR)), 2.51 (*m*, 4H, Ar-C*H*_2_-C_4_H_9_), 1.98–1.85 (*m*, 4H, Cy-*H*_2_), 1.72–1.43 (*m*, 10H, Cy-*H*_2_ und (C*H*_2_)_pentyl_), 1.33–1.25 (*m*, 8H, (CH_2_)_pentyl_), 0.86 (*t*, *J* = 6.7, 6H, (CH_3_)_pentyl_). ^13^C-NMR (125 MHz, CD_2_Cl_2_, 21 °C): *δ* = 164.8 (2C, C_Ar_-*C*H=N), 159.4 (2C, *C*_Ar_-OH), 145.6 (2C, α -*C*_Py_), 145.6 (2C, α-*C*_Py_), 145.4 (2C, γ -*C*_Py_), 135.5 (2C, *C*_Ar_-C_5_H_11_), 134.0 (2C, *C*_Ar_), 133.5 (2C, *C*_Ar_), 128.2 (4C, β-*C*_Py_), 120.8 (2C, *C*_Ar_-CH_2_-N_Py_), 118.9 (2C, *C*_Ar_-CHN), 71.7 (2C, *C*_Cy_HN), 60.4 (2C, Ar-*C*H_2_-Py), 35.0 (2C, C_Ar_-*C*H_2_-C_4_H_9_), 33.2 (2C, *C*_Cy_H_2_), 31.8 (2C, (*C*H_2_)_Pentyl_), 31.6 (2C, (*C*H_2_)_Pentyl_), 24.5 (2C, *C*_Cy_H_2_), 22.9 (2C, (*C*H_2_)_Pentyl_), 14.2 (2C, *C*H_3_). IR (solid): line graphic = 3396, 2925, 2854, 2561, 2374, 2186, 1967, 1627, 1472, 683. HRMS (ESI)*m/z*: Anal. Calcd. for [[C_42_H_54_BrN_4_O_2_]^+^]: 725.3413, found 725.3422. 

*(R*,*R)-(−)-N*,*N'-Bis(3-methyl-5-(pyridinium-1-ylmethyl)salicylidene)-1*,*2-cyclohexanediamine dibromide* (**14cA**) was prepared according to GP4 using **12cA** (2 eq., 6.5 mmol, 2.0 g) and (1*R*,2*R*)-(−)-1,2-diaminocyclohexane (1 eq., 3.2 mmol, 370 mg). The product was formed as a slightly yellow solid (2.7 mmol, 1.89 g, 84%). C_34_H_38_Br_2_N_4_O_2_, MW: 694.50 g/mol. M.p.: Decomp. at T > 200 °C. line graphic (*c* = 10 mg/mL, CH_2_Cl_2_) = −296.0. ^1^H-NMR (300 MHz, CD_2_Cl_2,_ 20 °C): δ = 14.09 (*b*, 1H, Ar-O*H*), 9.48 (*d*, *J* = 9.7, 4H, *o*-Py-*H*), 8.42 (*tt*, *J* = 7.9, *J* = 1.4, 2H, *p*-Py-*H*), 8.31 (*s*, 2H, Ar-C*H*=N-_Cy_), 7.96 (*t*, *J* = 7.2, 4H, *m*-Py-*H*), 7.84 (*d*, *J* = 2.2, 2H, Ar-*H*), 7.12 (*d*, *J* = 1.9, 2H, Ar-*H*), 6.08 (*q*, *J* = 13.7, Ar-C*H*_2_-Py), 3.42 (*m*, 2H, C_Cy_*H*N=CHR), 2.26 (*s*, 6H, C*H*_3_), 1.98–1.48 (*m*, 8H, (C*H*_2_)_Cy_). ^13^C-NMR (75 MHz, CDCL_3_, 21 °C): 164.8 (2C, *C*HN), 159.2 (2C, *C*_Ar_-OH), 145.6 (2C, γ-*C*_Py_), 145.6 (2C, α-Py-*C*), 145.5 (2C, α-Py-*C*), 136.1 (2C, *C*_Ar_), 134.1 (2C, *C*_Ar_-Me), 128.6 (2C, *C*_Ar_), 128.3 (4C, β-Py-*C*), 120.9 (2C, *C*_Ar_-CH_2_-Py), 118.9 (2C, *C*_Ar_-CHN), 71.8 (2C,*C*_Cy_HN), 60.1 (2C, Ar-*C*H_2_-Py), 33.1 (2C,*C*_Cy_H_2_), 24.5(2C,*C*_Cy_H_2_), 20.4 (2C, *C*H_3_). IR (solid): line graphic = 3381, 3123, 3044, 2928, 2857, 2054, 1627, 1476. MS (ESI) *m/z*: 613.2 (20%, M^+^), 597.1 (60%, M^+^–OH), 518.0 (100%, M^+^–Br), 454.2 (90%, M^+^–pyridine). HRMS (ESI) *m/z*: Anal. Calcd. for [[C_34_H_38_BrN_4_O_2_]^+^]: 613.2173, found: 613.2182.

*(R*,*R)-(−)-N*,*N'-Bis(3-tert-butyl-5-(4-methylpyridinium-1-ylmethyl)salicylidene)-1*,*2-cyclohexane-diamine dibromide* (**14aB**) was prepared according to GP4 using **12aB** (2 eq., 0.30 mmol, 109.3 mg) and (1*R*,2*R*)-(−)-1,2-diaminocyclohexane (1 eq., 0.15 mmol, 17.1 mg). The product was formed as a yellow solid (0.13 mmol, 107.3 mg, 89%). C_42_H_54_Br_2_N_4_O_2_, MW: 806.71 g/mol. M.p.: 198.2–200.3 °C (decomposition). line graphic (*c* = 6.4 mg/mL, CH_2_Cl_2_) = −286.1. ^1^H-NMR (500 MHz, CDCl_3_, 20 °C): δ =14.00 (*b*, 2H, Ar-O*H*), 9.31 (*d*, *J* = 6.4, 4H, *o*-Py-*H*), 8.37 (*s*, 2H, Ar-C*H*=N-Cy), 8.08 (*d*, *J* = 3.3, 2H, Ar-*H*), 7.78 (*d*, *J* = 6.3, 4H, *m*-Py-*H*), 7.28 (*d*, *J* = 2.3, 2H, Ar-*H*), 6.07–5.94 (*m*, 4H, Ar-C*H*_2_-Py), 3.42 (*m*, 2H, Cy-*H*N=CHR)), 2.59 (*s*, 6H, Py-C*H*_3_), 1.94–1.84 (*m*, 4H, Cy-*H*_2_), 1.67 (*m*, 2H, Cy-*H*_2_), 1.46 (*m*, 2H, Cy-*H*_2_), 1.25 (*s*, 18H, C(CH_3_)_3_). ^13^C-NMR (125 MHz, CDCl_3_, 20 °C): δ = 164.7, 158.9, 158.7, 144.4, 142.1, 132.7, 130.2, 128.4, 120.5, 118.0, 71.4, 59.4, 34.3, 33.1, 31.5, 24.1, 22.3. IR (solid): line graphic = 3387, 2946, 2862, 1628, 1469, 1280, 1224, 1149, 1019, 824, 787. MS (ESI) *m/z*: 725.3 (1%, [M]^+^−[Br]), 552.4 (7%, [M]^+^−[Py]), 459.3 (100%, [M]^+^−2×[Py]), 323.2 (1%, [M]^2+^), 276.7 (93%, [M]^2+^−[Py]). HRMS (ESI) *m/z*: Anal. Calcd. for [[C_42_H_54_N_4_O_2_]^2+^]: 323.2118, found: 323.2097.

*(R*,*R)-(−)-N*,*N'-Bis(3-pentyl-5-(4-methylpyridinium-1-ylmethyl)salicylidene)-1*,*2-cyclohexanediamine dibromide* (**14bB**) was prepared according to GP4 using **12bB** (2 eq., 0.30 mmol, 113.5 mg) and (1*R*,2*R*)-(−)-1,2-diaminocyclohexane (1 eq., 0.15 mmol, 17.1 mg). The product was formed as a beige-colored solid (0.14 mmol, 116.9 mg, 93%). C_44_H_58_Br_2_N_4_O_2_, MW: 834.76 g/mol. M.p.: 143.7–146.8 °C. line graphic line graphic (*c* = 9.5 mg/mL, CH_2_Cl_2_) = −289.1. ^1^H-NMR (300 MHz, CDCl_3_, 20 °C): δ = 13.97 (*b*, 2H, Ar-O*H*), 9.26 (*d*, *J* = 6.7, 4H, *o*-Py-*H*), 8.33 (*s*, 2H, Ar-C*H*=N-Cy), 7.78 (*m*, 2H, Ar-*H*), 7.76 (*m*, 4H, *m*-Py-*H*), 7.11 (*m*, 2H, Ar-*H*), 6.08–5.91 (*m*, 4H, Ar-C*H*_2_-Py), 3.41 (*m*, 2H, Cy-*H*N=CHR)), 2.59 (*s*, 6H, Py-C*H*_3_), 2.48 (*t*, *J* = 7.8, 4H, Ar-C*H*_2_C_4_H_9_), 1.93–1.84 (*m*, 4H, Cy-*H*_2_), 1.71–1.62 (*m*, 2H, Cy-*H*_2_), 1.55–1.43 (*m*, 6H, (C*H*_2_)_pentyl_ und Cy-*H*_2_), 1.29–1.19 (*m*, 8H, (C*H*_2_)_pentyl_), 0.81 (*t*, *J* = 6.8, 6H, C_4_H_9_C*H*_3_). ^13^C-NMR (125 MHz, CDCl_3_, 20 °C): δ =164.4, 159.0, 158.7, 144.3, 135.0, 133.6, 133.1, 128.4, 120.6, 118.4, 71.4, 59.2, 34.6, 33.0, 31.5, 31.3, 24.1, 22.5, 22.3, 14.1. IR (solid): line graphic = 3380, 2926, 2855, 1629, 1468, 1276, 1152, 1028, 829, 787. MS (ESI) *m/z*: 487.3 (5%, [M]^+^−2×[Py]), 337.2 (1%, [M]^2+^), 290.7 (100%, [M]^2+^−[Py]), 244.2 (8%, [M]^2+^−2×[Py]). HRMS (ESI) *m/z*: Anal. Calcd. for [[C_44_H_58_N_4_O_2_]^2+^]: 337.2274, found: 337.2298.

*(R*,*R)-(−)-N*,*N'-Bis(3-pentyl-5-(4-tert-butylpyridinium-1-ylmethyl)salicylidene)-1*,*2-cyclohexane-diamine dibromide* (**14bC**) was prepared according to GP4 using **12bC** (2 eq., 0.30 mmol, 126.1 mg) and (1*R*,2*R*)-(−)-1,2-diaminocyclohexane (1 eq., 0.15 mmol, 17.1 mg). The product was formed as a beige-colored solid (0.12 mmol, 113.1 mg, 82%). C_50_H_70_Br_2_N_4_O_2_, MW: 918.92 g/mol. M.p.: 154.7–157.3 °C. line graphic (*c* = 10.2 mg/mL, CH_2_Cl_2_) = −289.1. ^1^H-NMR (300 MHz, CDCl_3_, 20 °C): δ = 13.97 (*b*, 2H, Ar-O*H*), 9.44 (*d*, *J* = 6.7, 4H, *o*-Py-*H*), 8.34 (*s*, 2H, Ar-C*H*=N-Cy), 7.89 (*d*, *J* = 6.8, 4H, *m*-Py-*H*), 7.84 (*m*, 2H, Ar-*H*), 7.11 (*m*, 2H, Ar-*H*), 6.13–5.95 (*m*, 4H, Ar-C*H*_2_-Py), 3.42 (*m*, 2H, Cy-*H*N=CHR)), 2.48 (*t*, *J* = 7.8, 4H, Ar-C*H*_2_C_4_H_9_), 1.92–1.85 (*m*, 4H, Cy-*H*_2_), 1.72–1.61 (*m*, 2H, Cy-*H*_2_), 1.55–1.44 (*m*, 6H, (C*H*_2_)_pentyl_ and Cy-*H*_2_), 1.34 (*s*, 18H, Py-C(C*H*_3_)_3_), 1.29–1.19 (*m*, 8H, (C*H*_2_)_pentyl_), 0.81 (*t*, *J* = 6.8, 6H, C_4_H_9_C*H*_3_). ^13^C-NMR (125 MHz, CDCl_3_, 20 °C): δ = 170.6, 164.5, 158.9, 144.7, 135.2, 133.7, 133.1, 124.9, 120.7, 118.4, 71.5, 58.8, 36.5, 34.6, 33.1, 31.5, 31.3, 30.5, 30.0, 24.0, 22.5, 14.1. IR (solid): line graphic = 3380, 2927, 2857, 1630, 1459, 1275, 1167, 1109, 1025, 850, 815. MS (ESI) *m/z*: 379.3 (27%, [M]^2+^), 311.7 (100%, [M]^2+^−[Py]). HRMS (ESI) *m/z***:** Anal. Calcd. for [[C_50_H_70_N_4_O_2_]^2+^]: 379.2744, found: 379.2770.

*(R*,*R)-(−)-N*,*N'-Bis(3-pentyl-5-(3*,*5-dimethylpyridinium-1-ylmethyl)salicylidene)-1*,*2-cyclohexane-diamine dibromide* (**14bD**) was prepared according to GP4 using **12bD** (2 eq., 0.30 mmol, 117.8 mg) and (1*R*,2*R*)-(−)-1,2-diaminocyclohexane (1 eq., 0.15 mmol, 17.1 mg). The product was formed as a yellow solid (0.14 mmol, 117.9 mg, 91%). C_46_H_62_Br_2_N_4_O_2_, MW: 862.82 g/mol. M.p.: 139.3–141.2 °C. line graphic (*c* = 10.7 mg/mL, CH_2_Cl_2_) = −271.0. ^1^H-NMR (300 MHz, CDCl_3_, 20 °C): δ = 13.91 (*b*, 2H, Ar-O*H*), 9.13 (*m*, 4H, *o*-Py-*H*), 8.33 (*s*, 2H, Ar-C*H*=N-Cy), 7.92 (*m*, 2H, *p*-Py-*H*), 7.80 (*d*, *J* = 2.0, 2H, Ar-*H*), 7.08 (*d*, *J* = 2.0, 2H, Ar-*H*), 6.06–5.91 (*m*, 4H, Ar-C*H*_2_-Py), 3.43 (*m*, 2H, Cy-*H*N=CHR)), 2.54–2.47 (*m*, 16H, Ar-C*H*_2_C_4_H_9_ and Py-C*H*_3_), 1.93–1.85 (*m*, 4H, Cy-*H*_2_), 1.73–1.60 (*m*, 2H, Cy-*H*_2_), 1.55–1.43 (*m*, 6H, (C*H*_2_)_pentyl_ und Cy-*H*_2_), 1.29–1.20 (*m*, 8H, (C*H*_2_)_pentyl_), 0.82 (*t*, *J* = 6.8, 6H, C_4_H_9_C*H*_3_). ^13^C-NMR (125 MHz, CDCl_3_, 20 °C): δ = 164.5, 158.8, 146.0, 142.0, 138.3, 135.4, 133.7, 133.0, 120.6, 118.4, 71.6, 59.4, 34.6, 33.2, 31.5, 31.2, 24.1, 22.5, 18.7, 14.1. IR (solid): line graphic = 3386, 2924, 2855, 1627, 1468, 1277, 1171, 1030, 863, 783, 700. MS (ESI) *m/z*: 487.3 (52%, [M]^+^−2 × [Py]), 351.2 (34%, [M]^2+^), 595.4 (100%, [M]^2+^−[Py]). HRMS (ESI) *m/z*: Anal. Calcd. for [[C_46_H_62_N_4_O_2_]^2+^]: 351.2431, found: 351.2424.

*(R*,*R)-(−)-N*,*N'-Bis(3-pentyl-5-(quinolinium-1-ylmethyl)salicylidene)-1*,*2-cyclohexanediamine dibromide* (**14bE**) was prepared according to GP4 using **12bE** (2 eq., 0.30 mmol, 124.3 mg) and (1*R*,2*R*)-(−)-1,2-diaminocyclohexane (1 eq., 0.15 mmol, 17.1 mg). The product was formed as an orange solid (0.14 mmol, 127.9 mg, 94%). C_50_H_58_Br_2_N_4_O_2_, MW: 906.83 g/mol. M.p.: 146.2–147.8 °C. line graphic (*c* = 10.4 mg/mL, CH_2_Cl_2_) = −193.3. ^1^H-NMR (300 MHz, CDCl_3_, 20 °C): δ = 14.15 (*b*, 2H, Ar-O*H*), 10.32 (*d*, *J* = 5.5, 2H, quinoline-*H*), 9.17 (*d*, *J* = 8.3, 2H, quinoline-*H*), 8.59 (*d*, *J* = 8.9, 2H, quinoline-*H*), 8.39 (*s*, 2H, Ar-C*H*=N-Cy), 8.23 (*d*, *J* = 7.8, 2H, quinoline-*H*), 8.08 (*m*, 2H, quinoline-*H*), 7.83 (*m*, 2H, quinoline-*H*), 7.73 (*m*, 2H, quinoline-*H*), 7.43 (*m*, 2H, Ar-*H*), 7.11 (*m*, 2H, Ar-*H*), 6.48–6.36 (*m*, 4H, Ar-C*H*_2_-quinolinium), 3.47 (*m*, 2H, Cy-*H*N=CHR)), 2.59 (*s*, 6H, Py-C*H*_3_), 2.44 (*t*, *J* = 7.8, 4H, Ar-C*H*_2_C_4_H_9_), 1.96–1.84 (*m*, 4H, Cy-*H*_2_), 1.73–1.61 (*m*, 2H, Cy-*H*_2_), 1.54–1.38 (*m*, 6H, (C*H*_2_)_pentyl_ and Cy-*H*_2_), 1.28–1.12 (*m*, 8H, (C*H*_2_)_pentyl_), 0.77 (*t*, *J* = 6.8, 6H, C_4_H_9_C*H*_3_). ^13^C-NMR (125 MHz, CDCl_3_, 20 °C): δ = 164.5, 158.6, 150.8, 147.6, 137.9, 135.7, 133.7, 133.3, 132.9, 130.9, 129.9, 122.0, 119.5, 119.0, 118.5, 71.1, 56.6, 34.6, 32.9, 31.3, 31.2, 24.1, 22.4, 14.1. IR (solid): line graphic = 3385, 2924, 2854, 1626, 1593, 1526, 1465, 1373, 1230, 1020, 769. MS (ESI) *m/z*: 616.4 (10%, [M]^+^−[quinoline]), 487.3 (80%, [M]^+^−2×[quinoline]), 373.2 (14%, [M]^2+^), 308.7 (100%, [M]^2+^−[quinoline]). HRMS (ESI) *m/z*: Anal. Calcd. for [[C_50_H_58_N_4_O_2_]^2+^]: 373.2274, found: 373.2284.

*(R*,*R)-(−)-N*,*N'-Bis(3-pentyl-5-(4-(dimethylamino)pyridinium-1-ylmethyl)salicylidene)-1*,*2-cyclo- hexanediamine dibromide* (**14bF**) was prepared according to GP4 using **12bF** (2 eq., 0.30 mmol, 122.2 mg) and (1*R*,2*R*)-(−)-1,2-diaminocyclohexane (1 eq., 0.15 mmol, 17.1 mg). The product was formed as a yellow solid (0.14 mmol, 123.1 mg, 92%). C_46_H_64_Br_2_N_6_O_2_, MW: 892.85 g/mol. M.p.: 145.9–147.1 °C. line graphic (*c* = 10.2 mg/mL, CH_2_Cl_2_) = −269.1. ^1^H-NMR (300 MHz, CDCl_3_, 20 °C): δ = 13.91 (*b*, 2H, Ar-O*H*), 8.51 (*d*, *J* = 7.7, 4H, *o*-Py-*H*), 8.35 (*s*, 2H, Ar-C*H*=N-Cy), 7.73 (*d*, *J* = 1.9, 2H, Ar-*H*), 7.09 (*d*, *J* = 2.0, 2H, Ar-*H*), 6.89 (*d*, *J* = 7.8, 4H, *m*-Py-*H*), 5.50–5.37 (*m*, 4H, Ar-C*H*_2_-Py), 3.42 (*m*, 2H, Cy-*H*N=CHR)), 3.21 (*s*, 12H, Py-N(C*H*_3_)_2_), 2.47 (*t*, *J* = 7.8, 4H, Ar-C*H*_2_C_4_H_9_), 1.96–1.86 (*m*, 4H, Cy-*H*_2_), 1.74–1.59 (*m*, 2H, Cy-*H*_2_), 1.54–1.45 (*m*, 6H, (C*H*_2_)_pentyl_ and Cy-*H*_2_), 1.33–1.20 (*m*, 8H, (C*H*_2_)_pentyl_), 0.82 (*t*, *J* = 6.7, 6H, C_4_H_9_C*H*_3_). ^13^C-NMR (125 MHz, CDCl_3_, 20 °C): δ = 164.5, 158.4, 156.2, 142.6, 134.4, 133.4, 132.5, 121.5, 118.5, 108.0, 71.5, 56.0, 40.6, 34.7, 33.0, 31.5, 31.3, 24.1, 22.5, 14.1. IR (solid): line graphic = 3378, 2924, 2855, 1643, 1563, 1443, 1400, 1226, 1160, 1027, 823, 775. MS (ESI) *m/z*: 487.3 (38%, [M]^+^−2×[Py]), 366.3 (26%, [M]^2+^), 305.2 (100%, [M]^2+^−[Py]). HRMS (ESI) *m/z*: Anal. Calcd. for [[C_46_H_64_N_6_O_2_]^2+^]: 366.2540, found: 366.2536.

*(R*,*R)-(−)-N*,*N'-Bis(3-pentyl-5-(4-iodopyridinium-1-ylmethyl)salicylidene)-1*,*2-cyclohexanediamine dibromide* (**14bG**) was prepared according to GP4 using **12bG** (2 eq., 0.30 mmol, 147.1 mg) and (1*R*,2*R*)-(−)-1,2-diaminocyclohexane (1 eq., 0.15 mmol, 17.1 mg). The product was formed as a brown solid (0.14 mmol, 142.4 mg, 77%). C_42_H_52_Br_2_I_2_N_4_O_2_, MW: 1058.50 g/mol. M.p.: 221.2–223.7 °C (decomposition). line graphic (*c* = 9.6 mg/mL, DMSO) = −242.8. ^1^H-NMR (300 MHz, DMSO, 20 °C): δ = 14.19 (*b*, 2H, Ar-O*H*), 8.73 (*d*, *J* = 6.4, 4H, *o*-Py-*H*), 8.54 (*d*, *J* = 6.5, 4H, *m*-Py-*H*), 8.48 (*s*, 2H, Ar-C*H*=NCy), 7.47 (*b*, 2H, Ar-*H*), 7.20 (*b*, 2H, Ar-*H*), 5.69–5.60 (*m*, 4H, Ar-C*H*_2_-Py), 3.49 (*m*, 2H, Cy-*H*N=CHR)), 2.46 (*m*, 4H, Py-C*H*_2_C_4_H_9_), 1.93–1.86 (*m*, 4H, Cy-*H*_2_), 1.81–1.74 (*m*, 4H, (C*H*_2_)_pentyl_), 1.63–1.40 (*m*, 4H, Cy-*H*_2_), 1.33–1.19 (*m*, 8H, (C*H*_2_)_pentyl_), 0.84 (*t*, 6H, C_4_H_8_CH_3_). ^13^C-NMR (125 MHz, DMSO, 20 °C): δ = 165.4, 159.7, 144.0, 137.0, 134.7, 132.8, 132.7, 131.5, 121.1, 118.4, 117.4, 69.4, 58.9, 33.8, 32.2, 30.7, 30.4, 23.5, 21.8, 13.8. IR (solid): line graphic = 3396, 2923, 2852, 1615, 1441, 1160, 1026, 806. MS (ESI) *m/z*: 977.1 (1%, [M]^+^−[Br]), 4.87.3 (31%, [M]^+^−2×[Py]), 346.6 (100%, [M]^2+^−[Py]). HRMS (ESI) *m/z*: Anal. Calcd. for [[C_42_H_52_I_2_N_4_O_2_]^2+^]: 449.1084, found: 449.1105.

### 3.6. General Procedure for the Catalytic Asymmetric Synthesis of ***3*** (GP5)

To a solution of the salen ligand **14** (0.1 eq., 75 µmol) in CH_2_Cl_2_ (3 mL) a solution of Me_3_Al in toluene (2 M, 0.1 eq., 75 µmol, 38 µL) was added and the mixture was stirred for 3 h at ambient temperature. Afterwards aldehyde **1** (1 eq., 0.75 mmol), acylbromide **2** (6 eq., 4.5 mmol) and diisopropylethylamine (2.5 eq., 1.88 mmol) were added at −70 °C and the reaction mixture was stirred for 24 h at this temperature. The reaction was quenched by pouring into aqueous 1 M HCl (30 mL) and the product was extracted with CH_2_Cl_2_ (2 × 20 mL). The combined organic layers were dried over MgSO_4_ and filtered through a pad of silica gel. After removing the solvent *in vacuo* the desired *trans*-β-lactone **3** was obtained.

*(3S*,*4S)-trans-3-Methyl-4-(2-phenylethyl)-oxetan-2-one* (**3Aa**, 0.45 mmol, yield: 60%, *ee* = 90%, *dr* = 97:3) was prepared from propionyl bromide (**1A**) and 3-phenylpropionaldehyde (**2a**) according to GP5. The *dr* value was determined by ^1^H-NMR and the *ee* value by HPLC (Chiralcel OD-H, 97:3 *n*-hexane/ *i*PrOH, 1.0 mL/min, 210 nm). An analytically pure sample was obtained as a colorless oil by flash chromatography (pentane/diethyl ether 20:1). C_12_H_14_O_2_, MW: 190.24 g/mol. ^1^H-NMR (300 MHz, CDCl_3_, 20 °C): δ = 7.34–7.17 (*m*, 5H, Ar-*H*), 4.16 (*ddd*, *J* = 7.5, 5.9, 4.0, 1H, C*H*-O), 3.20 (*qd*, *J* = 7.5, 4.0, 1H, C*H*-C(O)), 2.77 (*m*, 2H, CH_2_-C*H*_2_-Ph), 2.13 (*m*, 2H, C*H*_2_-CH_2_-Ph), 1.32 (*d*, *J* = 7.5, 3H, C*H*_3_). The analytical data are in agreement with the literature [54].

*(3S*,*4S)-trans-3-Methyl-4-ethyloxetan-2-one* (**3Ab**, 0.44 mmol, yield: 59%, *ee* = 90%, *dr* = 98:2) was prepared from propionyl bromide (**1A**) and propanal (**2b**) according to GP5. The *dr* value was determined by ^1^H-NMR and the *ee* value by GC (Fisons Instruments HRGC Mega 2, Bondex-UN-β-column 20 m × 0.30 mm, 0.5 bar H_2_, method: 40 °C hold 5 min, ramp @ 2.5 °C/min till 100 °C, ramp @ 10.0 °C/min till 200 °C). An analytically pure sample was obtained as a colorless oil by flash chromatography (pentane/diethyl ether 20:1). C_6_H_10_O_2_, MW: 114.14 g/mol. ^1^H-NMR (300 MHz, CDCl_3_, 20 °C): δ = 4.13 (*td*, *J =* 6.6, 4.0, 1H, C*H*-O), 3.23 (*qd*, *J* = 7.5, 4.0, 1H, C*H*-C(O)), 1.97–1.72 (*m*, 2H, CH_3_-C*H_2_*-CH-O), 1.39 (*d*, *J* = 7.5, 3H, CH-C*H*_3_), 1.02 (*t*, *J* = 7.5, 3H, CH_2_-C*H*_3_). The analytical data are in agreement with the literature [54].

*(3S*,*4S)-trans-3-Propyl-4-(2-phenylethyl)-oxetan-2-one* (**3Ba**, 0.44 mmol, yield: 59%, *ee* = 95%, *dr* = 99:1) was prepared from valeroyl bromide (**1B**) and 3-phenylpropionaldehyde (**2a**) according to GP5. The *dr* value was determined by ^1^H-NMR and the *ee* value by HPLC (Chiralcel OD-H, 97:3 *n*-hexane/*i*PrOH, 1.0 mL/min, 210 nm). An analytically pure sample was obtained as a colorless oil by flash chromatography (CH_2_Cl_2_/pentane 3:1, then pentane/diethyl ether 40:1). C_14_H_18_O_2_, MW: 218.29 g/mol. ^1^H-NMR (300 MHz, CDCl_3_, 20 °C): δ =7.34–7.16 (*m*, 5H, Ar-*H*), 4.22 (*ddd*, *J =* 7.9, 5.0, 4.0, 1H, C*H*-O), 3.19 (*ddd*, *J* = 8.4, 6.9, 4.0, 1H, C*H*-C(O)), 2.75 (*m*, 2H, CH_2_-C*H*_2_-Ph), 2.11 (*m*, 2H, C*H*_2_-CH_2_-Ph), 1.70 (*m*, 2H, C*H_2_*-CH-C(O)), 1.41 (*m*, 2H, CH_3_-C*H*_2_-CH_2_), 0.92 (*t*, *J =* 7.3, 3H, C*H*_3_). The analytical data are in agreement with the literature [54].

*(3S*,*4R)-trans-3-Methyl-4-phenyloxetan-2-one* (**3Ac**, 0,18 mmol, yield 37%, *ee* = 96%, *dr* = 98:2) was prepared from propionyl bromide (**1A**) and benzaldehyde (**2c**) according to GP5, but using 0.49 mmol of aldehyde in 2 mL of CH_2_Cl_2_ and basic workup conditions. The reaction mixture was quenched with diisopropylethylamine (2 mL) and filtered through a short plug of Et_3_N-deactivated silica gel. CH_2_Cl_2_ was subsequently removed *in vacuo*. The crude mixture was purified by flash chromatography (Et_3_N-deactivated silica gel, ethyl acetate/petroleum ether 1:10). The *dr* value was determined by ^1^H-NMR and the *ee* value by HPLC (Chiralcel OD-H, 99:1 *n-*hexane/*i*PrOH, 0.5 mL/min, 210 nm). C_10_H_10_O_2_, MW: 162.18 g/mol. ^1^H-NMR (300 MHz, CDCl_3_, 20 °C): δ = 7.45–7.36 (*m*, 5H, Ar-*H*), 5.15 (*d*, *J =* 4.2, 1H, C*H*-O), 3.58 (*qd*, *J* = 7.5, 4.2, 1H, C*H*-C(O)), 1.53 (*d*, *J* = 7.5, 3H, C*H*_3_). The analytical data are in agreement with the literature [54].

Preformation of the catalyst ***{***[1,1'-[(1R,2R)-1,2-cyclohexandiylbis[imino-к2N[5-pentyl-2-(hydroxy-к2O)-3,1-phenylen]*methylen]bis*[4-methyl-pyridinium]*](2-)]-methylaluminum(III)}dibromide* (**4bB**): To a solution of ligand **14bB** (0.13 g, 0.16 mmol, 1.0 equiv.) in CH_2_Cl_2_ (3.0 mL) a solution of Me_3_Al in toluene (2 M, 0.10 mL, 0.16 mmol, 1.0 equiv.) was added. The mixture was stirred for 3 h at ambient temperature. The reaction mixture was poured into 20 mL of pentane to precipitate complex **4bB**. Subsequently the mixture was centrifuged and the supernatant removed. Washing the catalyst with additional pentane (10 mL) and drying *in vacuo* afforded the active catalyst as orange powder in quantitative yield. C_45_H_59_AlBr_2_N_4_O_2_, MW: 874.77 g/mol. ^1^H-NMR (500 MHz, CD_2_Cl_2_, 20 °C): δ = 9.48 (*m*, 2H, *o*-Py-*H*), 9.42 (*m*, 2H, *o*-Py-*H*), 8.49 (*m*, 1H, Ar-C*H*=N-Cy), 8.15 (*m*, 1H, Ar-C*H*=NCy), 7.78 (*m*, 1H, *m*-Py-*H*), 7.74–7.71 (*m*, 3H, *m*-Py-*H*), 7.53 (*d*, *J* = 5.5, 2H, Ar-*H*), 7.28 (*m*, 1H, Ar-*H*), 7.24 (*m*, 1H, Ar-*H*), 6.52 (*d*, *J* = 13.7, 1H, Ar-C*H*_2_-Py), 6.38 (*d*, *J* = 13.5, 1H, Ar-C*H*_2_-Py), 5.98 (*d*, *J* = 13.7, 1H, Ar-C*H*_2_-Py), 5.86 (*d*, *J* = 13.5, 1H, Ar-C*H*_2_-Py), 3.63 (*m*, 1H, Cy-*H*N=CHR), 3.15 (*m*, 1H, Cy-*H*N=CHR), 2.61–2.45 (*m*, 11H, Cy-*H*_2_ and (C*H*_2_)_pentyl_ and Py-C*H*_3_), 2.34 (*m*, 1H, Cy-*H*_2_), 2.05 (*m*, 2H, Cy-*H*_2_), 1.66–1.58 (*m*, 4H), 1.52–1.45 (*m*, 4H), 1.39–1.26 (*m*, 8H, (C*H*_2_)_pentyl_), 0.93–0.87 (*m*, 6H, (C*H*_2_)_pentyl_), −1.31 (*s*, 3H, Al-C*H*_3_). ^13^C-NMR (125 MHz, CD_2_Cl_2_, 20 °C): δ = 168.8, 162.1, 161.3, 160.7, 158.7, 158.4, 144.6, 144.5, 138.5, 136.9, 135.5, 134.5, 131.9, 131.3, 127.9, 127.7, 124.9, 124.3, 119.2, 119.1, 66.2, 62.5, 60.6, 60.1, 34.4, 31.5, 31.4, 31.2, 31.2, 29.7, 26.9, 24.1, 23.7, 22.5, 22.3, 21.9, 13.9, 13.8. MS (ESI) *m*/*z*: 793.4 (100%, [M]^+^–Br) 700.3 (86%, [M]+–Py–Br). HRMS (ESI) *m*/*z*: Anal. Calcd. for [[C_45_H_59_AlBrN_4_O_2_]^+^]: 793.3631, found: 793.3620.

## 4. Conclusions

In summary, we have reported a catalyst which offers the highest enantio- and *trans*-selectivity known so far for the catalytic asymmetric synthesis of β-lactones by [2+2] cyclocondensation of acyl halides and aldehydes. Catalysts for the asymmetric formation of *trans*-β-lactones are of major interest, since *trans*-β-lactones offer a divergent and atom-economic access to the important class of *anti*-aldol products. In our catalyst, an Al-center (offering a single coordination site) cooperates with a picolinium bromide moiety based on our recently published strategy to combine the concepts of Lewis acid and organic aprotic ion pair catalysis in a single catalyst system. Since cationic residues like pyridinium units have been found to be essential for both high *trans*- and enantioselectivity (suggesting that the positive charge enables an ion pair catalysis pathway), we have investigated the question, if substituents on the pyridinium rings can be utilized to further improve the catalyst efficiency, as they might display a significant impact on the effective charges. In the present study we have thus compared a small library of aluminum-salen/bispyridinium catalysts, mainly differing in the substituents on the pyridinium rings. NBO calculations have revealed though that the different catalyst efficiencies can arguably not be explained by the variation of the effective charges, since there are only very small differences for σ-donor or σ-acceptor substituted pyridinium systems. However, we have noticed that the substituents have a major impact on the catalyst stability and presumably they have also an impact on the reactive conformation of the proposed acyl halide enolate intermediates.
